# A novel therapeutic approach to colorectal cancer in diabetes: role of metformin and rapamycin

**DOI:** 10.18632/oncotarget.26641

**Published:** 2019-02-12

**Authors:** Alice Gerges Geagea, Manfredi Rizzo, Abdo Jurjus, Francesco Cappello, Angelo Leone, Giovanni Tomasello, Céline Gracia, Sahar Al Kattar, Liliane Massaad-Massade, Assaad Eid

**Affiliations:** ^1^ Department of Internal Medicine, University of Palermo, Palermo, Italy; ^2^ Department of Anatomy, Cell Biology and Physiological Sciences, Faculty of Medicine, American University of Beirut, Beirut, Lebanon; ^3^ Equipe Nouvelles Thérapies Anticancéreuses, UMR8203 CNRS, Gustave Roussy, Villejuif, France; ^4^ Department of Biomedicine, Neurosciences and Advanced Diagnosis, School Of Medicine of Palermo, Palermo, Italy

**Keywords:** colorectal cancer, diabetes mellitus, probiotics, inflammatory cytokines, mTOR

## Abstract

The link between colorectal cancer (CRC), diabetes mellitus (DM) and inflammation is well established, and polytherapy, including rapamycin, has been adopted. This study is a novel approach that aimed at assessing the effect of a combination therapy of metformin and rapamycin on the control or prevention of CRC in diabetic animals, in presence or absence of probiotics.

Fifty NOD/SCIDs male mice developed xenograft by inoculating HCT116 cells. They were equally divided into diabetics (induced by Streptozotocin) and non-diabetics. Metformin was given in drinking water, whereas rapamycin was administered via intra-peritoneal injections. Probiotics were added to the double therapy two weeks before the sacrifice. Assessment was performed by clinical observation, histological analysis, Reactive oxygen species (ROS) activities and molecular analysis of Interleukin 3 and 6, Tumor Necrosis Factor alpha, AMP-activated protein Kinase and the mammalian target of rapamycin. Decreases in the level of tumorigenesis resulted, to various extents, with the different treatment regimens. The combination of rapamycin and metformin had no significant result, however, after adding probiotics to the combination, there was a marked delay in tumor formation and reduction of its size, suppression of ROS and a decrease in inflammatory cytokines as well as an inhibition of phosphorylated mTOR.

Existing evidence clearly supports the use of rapamycin and metformin especially in the presence of probiotics. It also highlighted the possible mechanism of action of the 2 drugs through AMPK and mTOR signaling pathways and offered preliminary data on the significant role of probiotics in the combination. Further investigation to clarify the exact role of probiotics and decipher in more details the involved pathways is needed.

## INTRODUCTION

Several investigators, including our team, reported the co-occurrence of diabetes mellitus (DM) and colorectal cancer (CRC) along with bowel inflammation and dismicrobism [1–6]. Moreover, multiple reports suggested the involvement of the gut microbiome in the evolution of DM, and that potential modulation of the intestinal microbiota could prevent or delay its progression [7]. Furthermore, data are increasing about the greater risk for CRC in patients with DM by almost 1.2% to 1.5% [8]. According to the Global Burden of Disease study data, mortality from CRC increased annually from 1990 through 2013 in line with a worldwide decrease in the age of onset of DM [9]. In addition, Type 2 diabetes mellitus (T2DM) has been reported to increase the risks of a wide spectrum of cancers including CRC, and that 14% of CRC patients have T2DM as a comorbid condition at diagnosis [10]. They conferred an increased risk of CRC in T2DM patients and a higher mortality rate [6, 9, 11–14]. In addition, CRC, colorectal adenoma and chronic colitis are positively associated with inflammation, T2DM and hyperinsulinemia, thus representing the link between the various disease entities [15]. Further studies have also shown that in human epithelial colorectal cancer cells, high glucose or insulin activates a cascade of cross reacting pathways leading to an alteration in a panoply of proteins in the signaling cascade involved in cell proliferation, survival and apoptosis [15].

Moreover, it is also well documented that in diabetes and CRC, there is an increased generation of Reactive Oxygen Species (ROS). More importantly, in tumors, RO metabolites can act as signaling molecules to promote cell survival over apoptosis. On the other hand, studies have also shown that in diabetes, there is an increased production of 20-hydroxyeicosatetraenoic acid (20-HETE) resulting from arachidonic acid cytochrome P450-dependent metabolism. 20 HETE contributes, through a ROS dependent pathway, to organ damage and plays a role in inflammatory responses, carcinogenesis, cardiac functions and vascular hypertrophy among others [16, 17]. So we hypothesize that inhibitors of 20-HETE synthesis might have anticancer and anti-diabetic activities. However, the upstream and downstream signaling pathways leading to injury are not yet fully studied and defined. The mechanistic pathway can be simplified by inactivating AMP-activated protein Kinase (AMPK), activating the mammalian target of rapamycin (mTOR) signaling pathway, and consequently increasing tumor development.

It is also important to note that chronic inflammation, as a process, forms a favorable environment for such a mechanistic pathway to occur. It involves a balance between a huge panel of bioactive molecules, pro and anti-inflammatory provided from resident or infiltrating inflammatory cells [6]. However, a persistent or an inadequately resolved chronic inflammation tilts the balance in favor of pro-inflammatory agents, may increase the risk of several pathologies such as IBD, CRC and T2DM.

In case of co-occurrence of diabetes and cancer, inflammation is characterized by an upregulation of inflammatory cytokines, mainly IL-6, IL-1 and TNF α, as well as TGFβ, NFKB, and ROS among others. These molecules are reported to be powerful tumor promoters, which create a favorable environment for malignancies, genomic instability, oxidative stress and angiogenesis. All of these phenomena are key players in linking inflammation to carcinogenesis and other systemic diseases like diabetes [6].

Pharmacologically modulating the inflammatory process might be of value in decreasing, preventing or even managing the process underlying these diseases [6].

Metformin, an oral biguanide, discovered almost a hundred years ago, is prescribed to over 120 million people worldwide for the treatment of conditions including T2DM, polycystic ovarian syndrome, and gestational diabetes [18]. Over the past decade, multiple epidemiologic, preclinical and clinical studies have consistently associated metformin with decreased cancer incidence and cancer-related mortality, shedding light on the anti-cancer effects of this hypoglycemic agent [6, 19]. Although the exact mechanisms of metformin action are not entirely understood, there is a robust literature that defines the hallmarks of its cellular and molecular signaling in colon cancer cell lines with regards to AMPK activation that leads to inhibition of mTOR and a reduction in translation initiation, thus providing a possible role of metformin in the inhibition of cancer cell growth [20, 21].

Similarly, rapamycin, discovered more than thirty years ago as an immunosuppressor, has been used successfully to reduce organ rejection with kidney transplantation [22]. Furthermore, rapamycin inhibited cell growth in tumor cell lines including CRC cell lines like Caco2, HT29 [23], which involves binding to the mammalian target of rapamycin (mTOR) whose signaling pathway is critical to cell growth, proliferation, and survival; in brief, rapamycin could inhibit most of these hallmark processes of cancer [23, 24].

Exploring the possible additive effects of metformin (an AMPK activator) and rapamycin (a blocker for mTOR activation) might open a new horizon in dealing with the two co-morbid disease entities. Furthermore, modulation of the microbiota by increasing its diversity through probiotic use might hold the promise of effective protection against both DM and CRC [25]. The aim of this study was to determine the roles of metformin and rapamycin, alone and in combination in the management of diabetes and colorectal cancer in an ectopic xenografts mouse model, at clinical, histological and molecular levels, with an emphasis on the downstream signaling elicited by these drugs in the presence of probiotics.

## RESULTS

### Clinical Profile

Mice in group 1 (G1) (controls, non-treated, having the HCT116 cells xenograft) had the worst clinical profile. Two mice had diarrhea and rectal bleeding as well as weakness and low alertness. In addition, one mouse died two weeks before the sacrifice time.

On the other hand, groups treated with metformin, with or without rapamycin, had a better clinical profile when compared to the mice in G1; however, there were no significant changes in stools, and activity. Besides, animals treated with probiotics in addition to rapamycin and metformin had the best clinical profile, they had no diarrhea, and good body conditions. These two groups had the lowest DAI as shown in Figure 2. It is also noteworthy that there was a trend of decreased body weight in non-treated G1 mice, but the variations were not significant. All mice in the other groups showed a gradual increase in body weight without significant differences.

**Figure 1:**
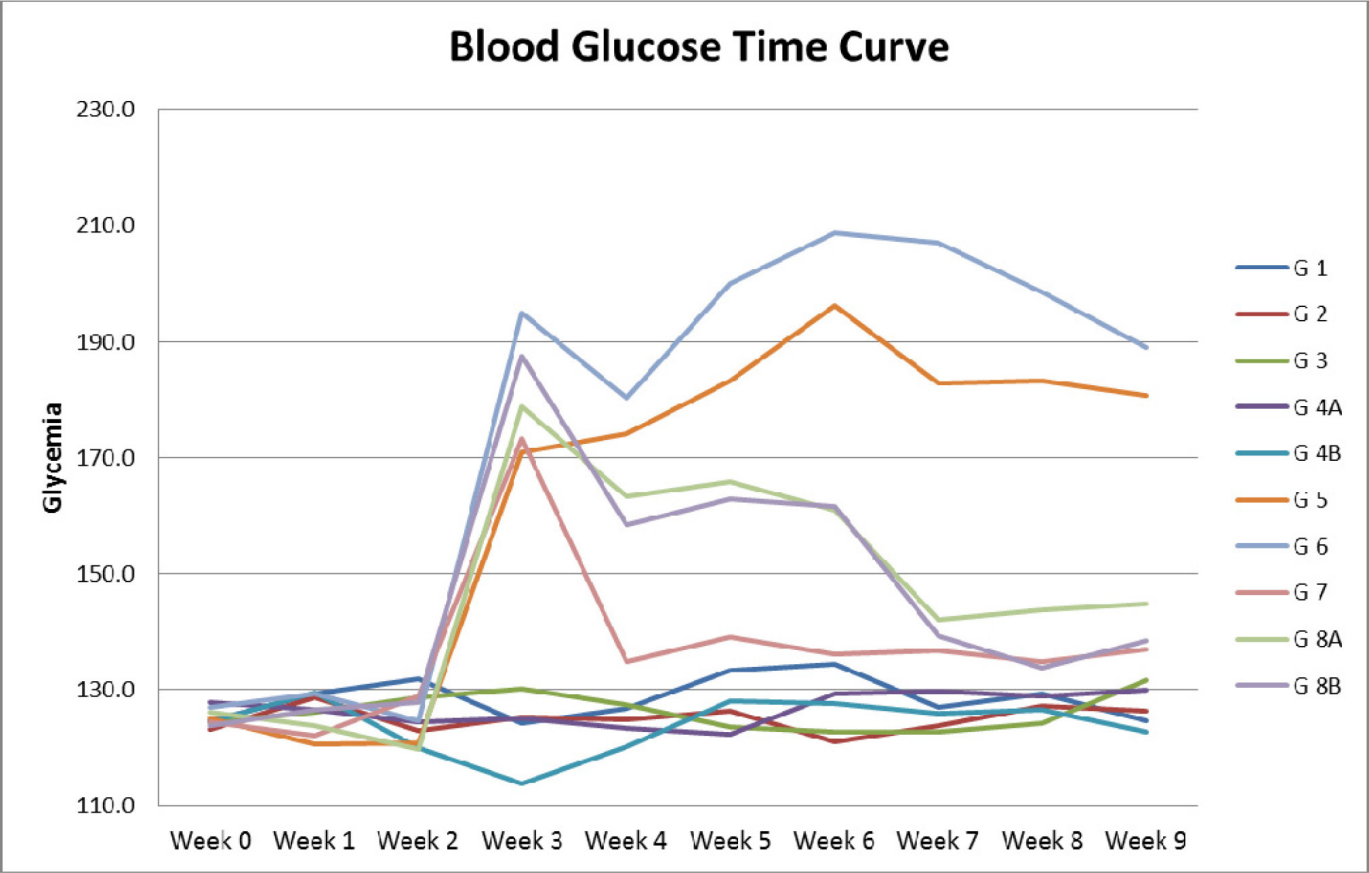
Blood glucose time curve Note the difference in Glycemia levels between diabetic and non-diabetic groups, as well as the drop in glycemia in diabetic animals in groups 7, 8A and 8B treated respectively with metformin alone, metformin and rapamycin, probiotics with metformin and rapamycin.

**Figure 2:**
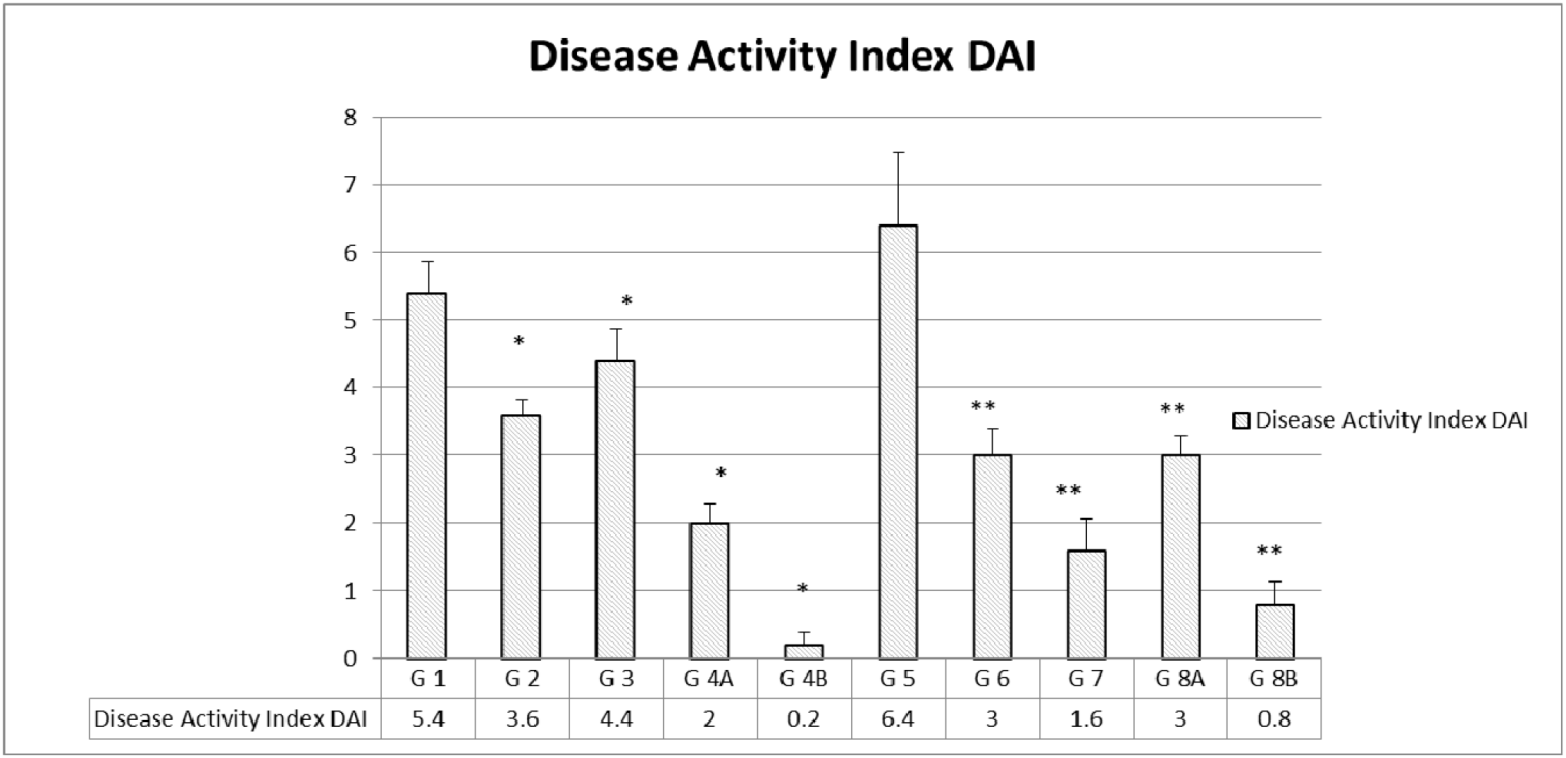
Disease activity index (DAI) in the different groups The values represent mean ± SEM (n = 6). Significance of p<0.05 was indicated by (^*^) and (^**^) when compared to diabetic control and non-diabetic control respectively.

Diabetes induction was successful in all animals injected with STZ; they had glycemia levels higher than 150 mg/dl even 10 days after injection (e.g. 128.52 in non-diabetics (ND) G1 and 163.72 in diabetics (D) G5). Treatment with metformin was able to reduce glycemia levels at all time points, (e.g. G3 and G7 had average glycemia levels of 126.6 vs 136.6 mg/dl, respectively).

As expected, rapamycin did not show any glucose lowering effect in both ND and D animals (ND- G2 124.96 vs ND-G1 128.52, p>0.05 and D-G6 175.88 vs D-G5 163.72 p>0.05). Moreover, adding a combination of metformin and rapamycin did not produce any significant added effect in lowering glucose below the metformin level alone (*e.g.* ND-G4A 124.38 vs ND-G3 126.18, p>0.05 and D-G8A 146.90 vs D-G7 136.68, p>0.05), Figure 1.

Moreover, probiotics added to metformin and rapamycin did not exhibit any additive effect in decreasing the glucose levels in the sera of animals. In brief, metformin alone normalized the glucose levels with no added effect from rapamycin and probiotics.

Disease Activity Index (DAI) which included multiple parameters was assessed on a regular basis, as described before, and a total of none was added for the highest disease activity. As expected, the highest indices were encountered in the non-treated groups in both D G5 (6.4) and ND G1 (5.4). However, ND animals treated with rapamycin alone G2 (3.6) or metformin alone G3 (4.4) had a lower DAI. As for the combination treatment, there was a limited additive effect in the ND G4A (2) compared to a lack of such an effect in the diabetics G8A (3).

On the other hand, when the combination of rapamycin and metformin was supplemented with probiotics, the DAI decreased drastically and significantly in both ND 4B (0.2) and D G8B (0.8), (Figure 2).

### Tumor frequency and volume

All mice injected with the -HCT116 cells developed tumors in their right flank (site of HCT116 injection), except for 3 groups; group 4A treated with metformin and rapamycin where 4 only out of 5 mice had tumors, and in groups 4B and 8B, where probiotics were added, tumor formation decreased by 40% as it occurred in only 3 out of 5 animals with a significantly smaller size.

**Figure 3:**
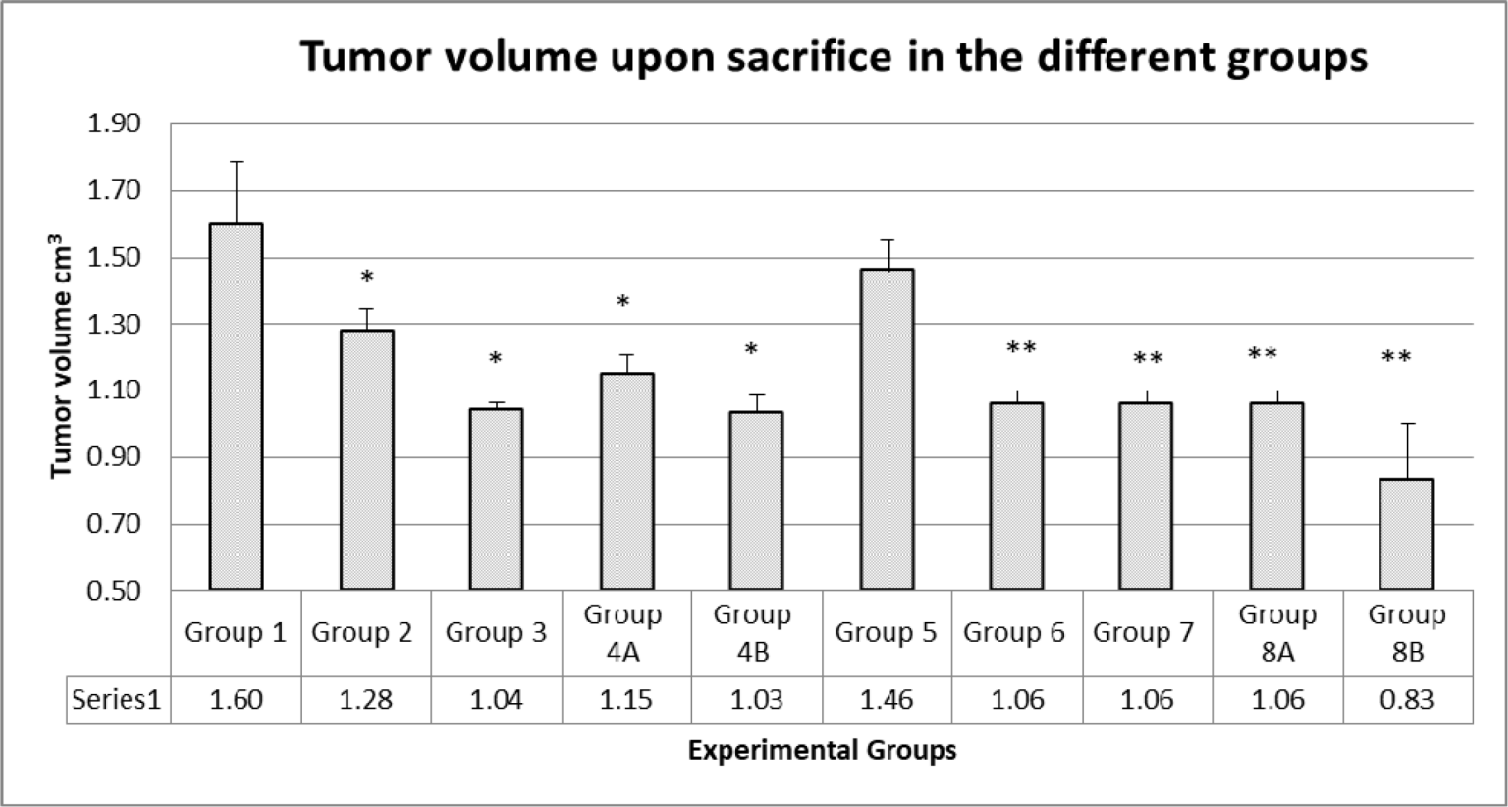
Tumor volumes upon sacrifice There was no additive effect of the combination therapy. In diabetics, group 8B, the probiotics with the combination had a significant antitumor effect. The values represent mean ± SEM (n = 6). Significance of p<0.05 was indicated by (^*^) and (^**^) when compared to diabetic control and non-diabetic control respectively.

Concerning tumor onset, a delay in tumor formation was observed in groups treated with metformin and rapamycin plus or minus probiotics, when compared to non-treated G1 mice. In G1 (non-treated) tumor appeared only 7 days after HCT116 injection; In contrast, in G8B treated with rapamycin, metformin and probiotics, tumor formation was delayed till day 15 by 88% and in 8A till day 14, respectively (Table 1), with significantly smaller size (Figure 3).

**Table 1:** Frequency and date of tumor formation

Group	Treatment	Tumors appeared after	Number of animals
**1**	Non Diabetic Non-treated	7 days	5 out of 5
**2**	Non Diabetic rapamycin	9 days	5 out of 5
**3**	Non Diabetic Met	9 days	5 out of 5
**4A**	Non Diabetic Met+ rapamycin	14 days	4 out of 5
**4B**	Non Diabetic Met + rapamycin + probiotics	14 days	3 out of 5
**5**	Diabetic Non-treated	9 days	5 out of 5
**6**	Diabetic rapamycin	10 days	5 out of 5
**7**	Diabetic Met	10 days	5 out of 5
**8A**	Diabetic Met + rapamycin	14 days	5 out of 5
**8B**	Diabetic Met + rapamycin + probiotics	15 days	3 out of 5

**Figure 4:**
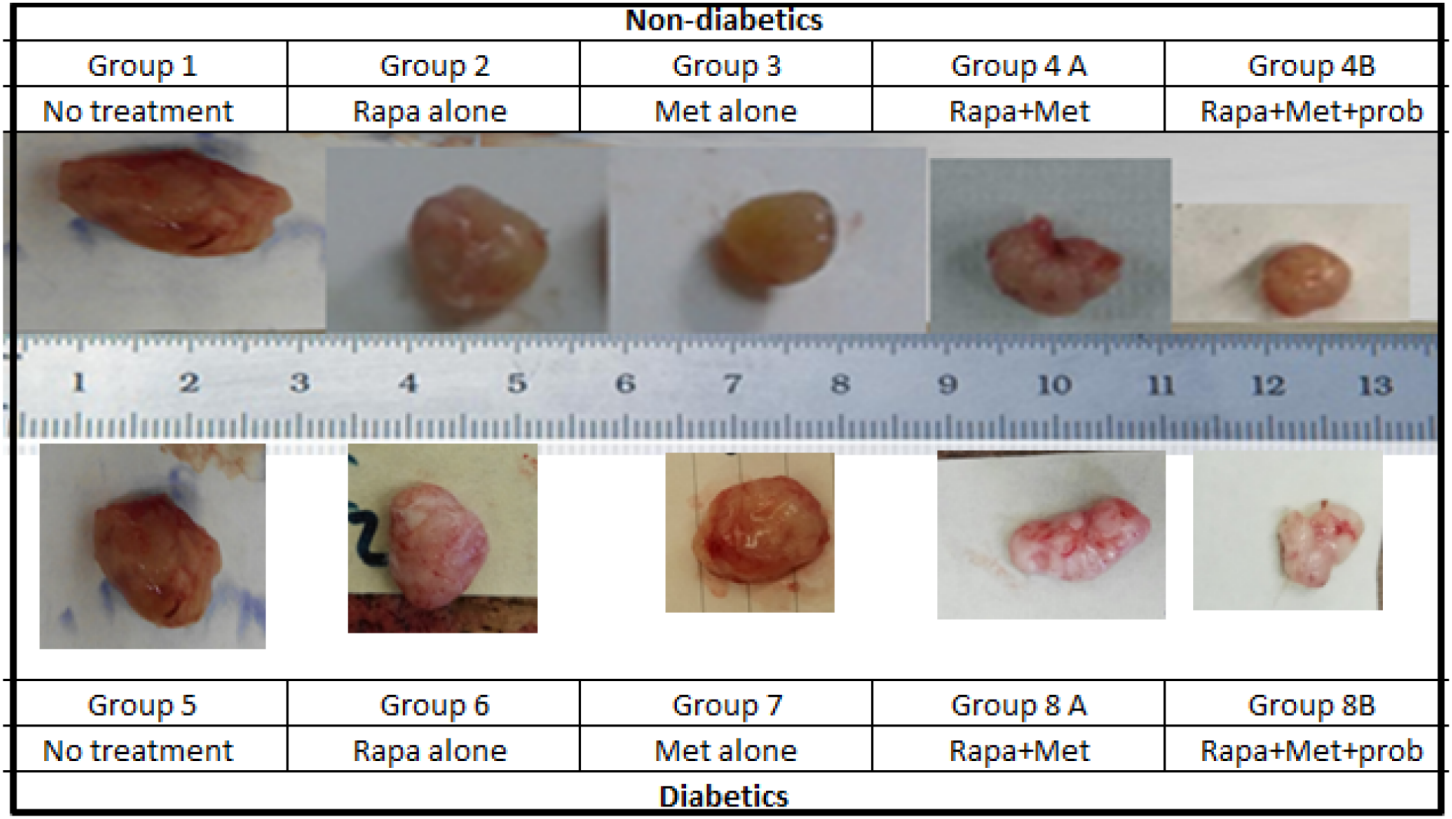
Prototype of tumors upon sacrifice, formed in non-diabetic and diabetic mice treated with rapamycin, metformin and their combination with probiotics Note the difference in tumor size in the different groups; animals from groups 4B and 8B treated with rapamycin and metformin in combination with probiotics had significantly smaller tumor size when compared to groups treated with Met alone, rapamycin alone or untreated animals.

The results showed that the highest tumor volumes were obtained in non-treated mice (groups 1 and 5, 1.6 and 1.45 cm^3^, respectively). In groups taking rapamycin alone or metformin alone, there was a reduction in tumor volume of 20% and 35%, respectively (G2 with rapamycin 1.28 and G3 with metformin 1.04 cm^3^). For groups taking the combined therapy metformin and rapamycin, G4A and G8A had also significantly small tumor volumes (G4A 1.15 and G8A 1.06 cm^3^) close to volumes from metformin alone or rapamycin alone; obviously there was no added effect of the 2 drugs. However, groups taking probiotics, G4B and G8B, had significantly the lowest tumor volumes (1.03 and 0.83 cm^3^, respectively) at all time points, with a decrease in tumor volume of about 36% and 43%, respectively (Figures 3 and 4).

### Histological alterations

Histological studies performed on the liver and kidneys showed no signs of toxicity. On the other hand, the histopathology of the xenograft was evaluated. It showed a wide range of alterations in the xenograft growth and morphology in the various groups. Treatment of ND mice with metformin alone, rapamycin alone or with the combination plus probiotics, led to various degrees of necrosis in the tumor xenograft. The most pronounced growth decrease and necrosis were in the presence of probiotics. Compared to the non-treated mice, those treated had a smaller size tumor, much less of inflammatory cells, and a lower density of tumor cells. The non-treated showed also some ascites fluid within the well circumscribed tumor and less vascularity.

**Figure 5:**
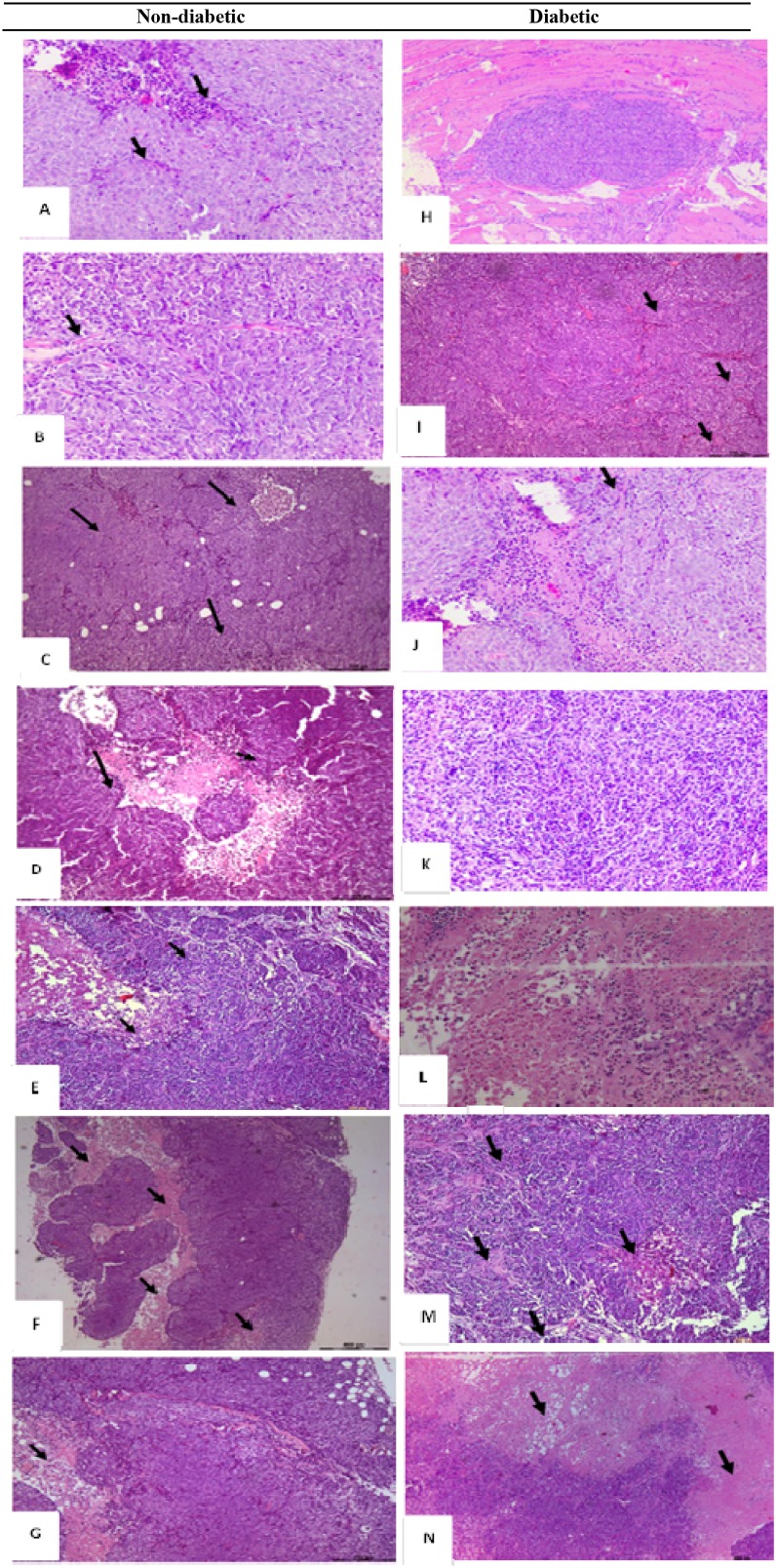
H&E histological examination of the representative HCT116 xenograft tumors in the different groups **(A)** a 200x magnification of a tumor section in non-diabetic, injected with HCT116 cells and non-treated showing high cellular density, vascularization (black arrows), and tumor cells surrounded by a remarkable infiltration of inflammatory cells (red arrows). **(B)** a 20x magnification showing some necrotic areas in the tumor (Black arrows) in non-diabetics, rapamycin alone (G2) **(C)** 20x magnification showing some necrotic areas in the tumor (Black arrows) in non-diabetics metformin alone **(D)** 4x and **(E)** 200x magnification show large necrotic areas in the tumor section with low cell density (black arrows) in non-diabetics treated with metformin combined to rapamycin. **(F)** 40x and **(G)** 200x magnification showing necrotic areas (Black arrows), along with a lower density of the cells in diabetic treated with metformin, rapamycin combined with probiotics (G4B). Note that all tumors from 5 animals in the same group showed similar morphology. **(H)** Whole view of a well-demarcated tumor formed with a scanty fibrous capsule and a moderately produced connective tissue in diabetic non-treated animals. Note the sheet-like proliferation showing growth of solid tumor cells. **(I)** 200x magnification of the tumor section in G5, note the high density of the cells along with increase in vascularity (black arrows) **(J)** 200x magnification of a tumor section, showing a moderate cells density along with an increase in vascularity (black arrows) in diabetics treated with rapamycin alone (G6). **(K)** 200x magnification of a tumor section, note the moderate density of the cells in diabetics treated with metformin alone (G7). **(L)** 200x magnification of tumor section from diabetic mice treated with metformin and rapamycin showing a lesser density of the cells than either alone **(M)** 200x and **(N)** 20x magnification of tumor section from diabetic mice with the triple therapy showing necrotic areas (Black arrows), along with a significant decrease in cellular density.

Moreover, in diabetic animals, the same picture and trend prevailed with a much lower density of cells and more of necrosis in the combination treated mice especially with probiotics. However, it is worth noting that metformin and rapamycin did not exhibit an additive inhibitor effect, yet the density of the tumor cells was relatively lower, and the ascites fluid was also less. The same findings were consistent in all the animals of a given group (Figure 5A–5N).

#### Descending colon tissues

**Figure 6:**
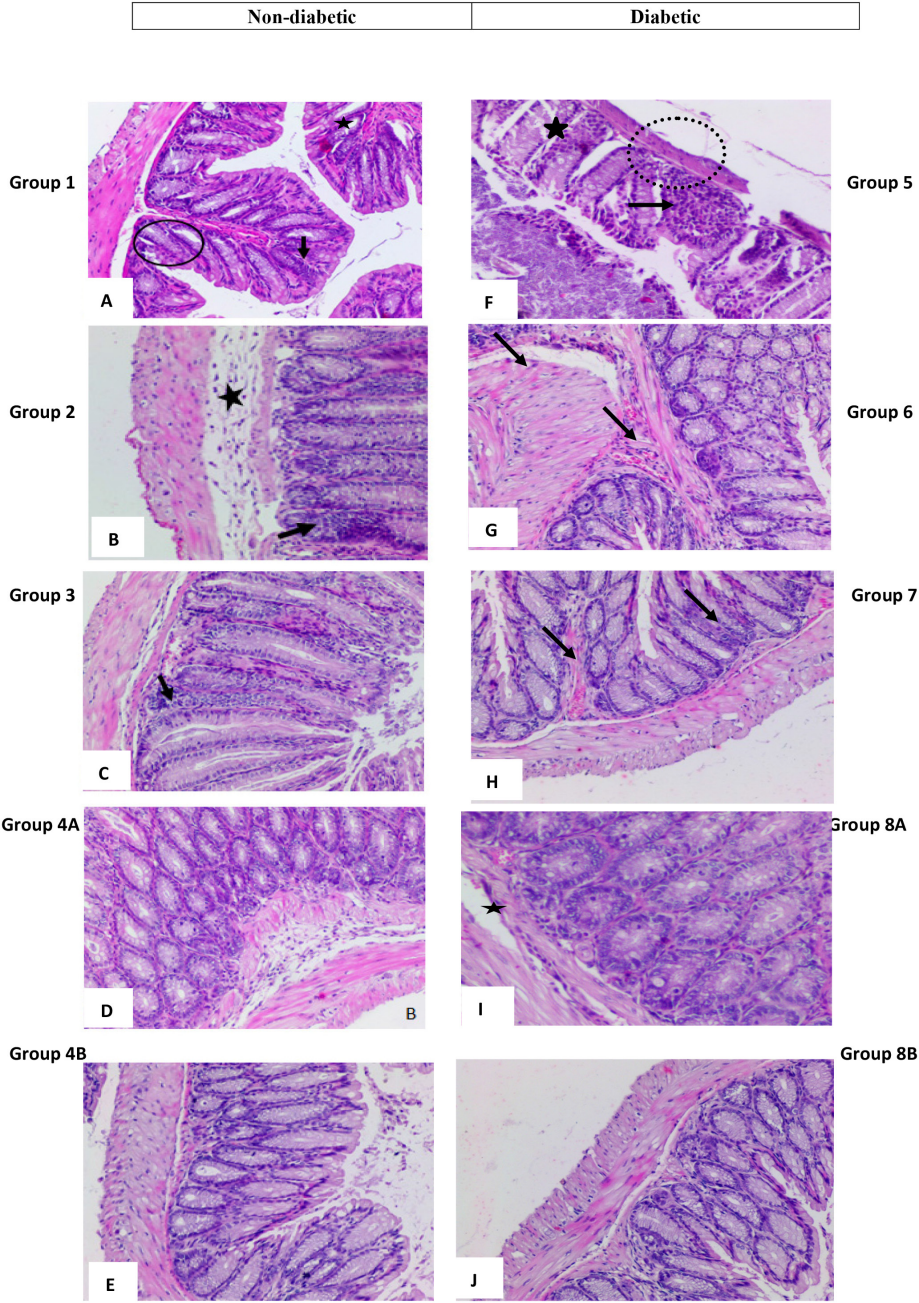
High power magnification of H&E stained colonic tissue obtained from the different groups **(A** and **F)** represent colon sections from non-treated animals, respectively non-diabetic and diabetic showing a marked hyperplasia with loss of goblet cells (black circles), polyp formation (star shape) and inflammatory cells infiltration (black arrows) seen in animals as well as thinning of the colonic layers (dotted circle) and extensive crypt dysregulation (star shape). **(B** and **G)** animals treated with rapamycin alone in non-diabetic (G2) and diabetic (G7), respectively show few inflammatory cell aggregates (black arrows), in addition to some dysregulation in epithelial cell lining and the sub-mucosal edema (star shape). Colon sections in **(C** and **H)** from animals treated with metformin alone, in non-diabetics and diabetics, respectively, showing few inflammatory cell aggregates (black arrows) and a close to normal colonic structure. **(D** and **I)** show normal colonic structure and normal goblet cell distribution, in addition to a moderate and sub-mucosal edema (star shape) in non-diabetic and diabetic animals treated with metformin and rapamycin. An almost normal colonic structure is seen in animals treated with rapamycin probiotics and metformin as in **(E** and **J)**.

**Figure 7:**
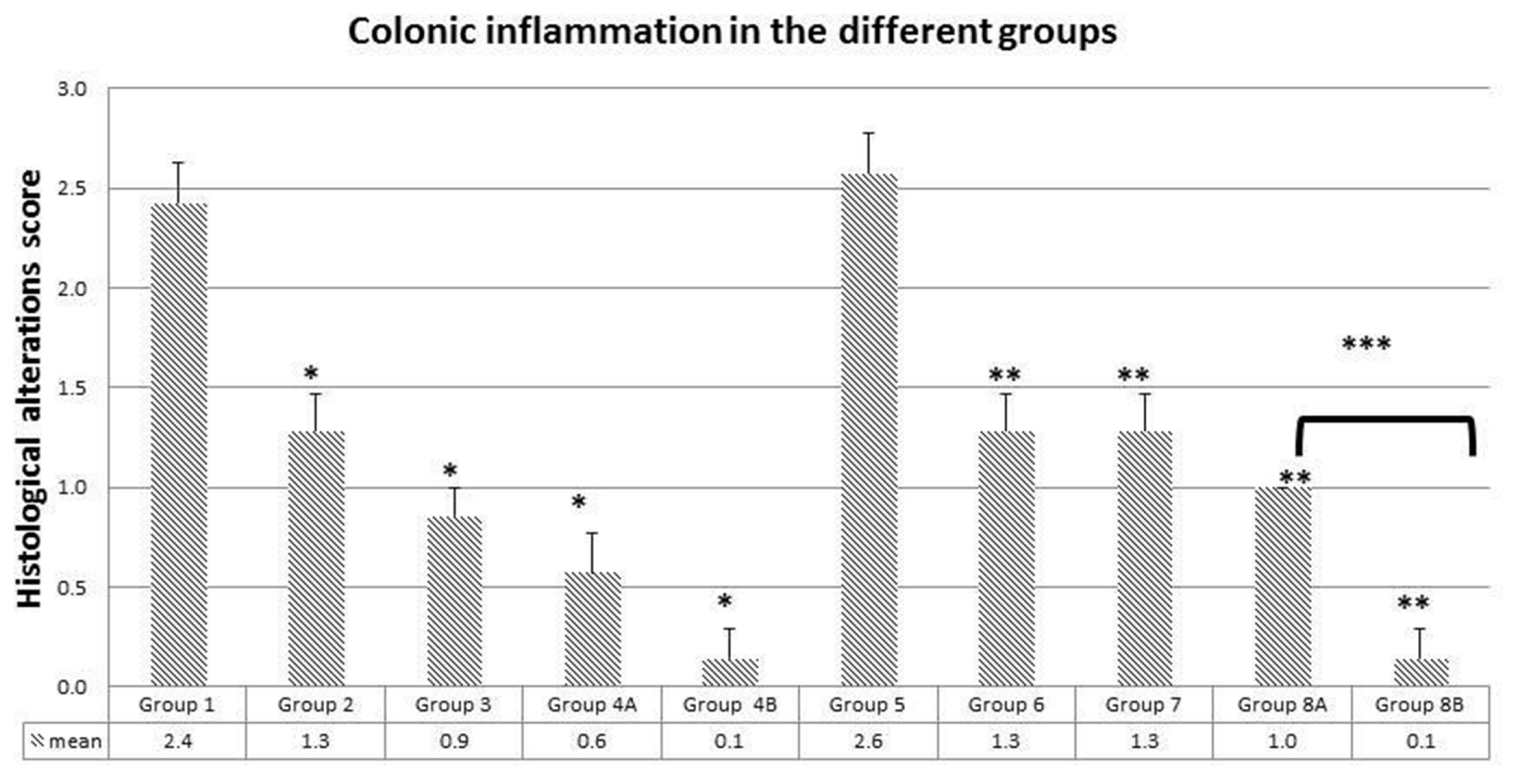
Colonic inflammation average in the different groups Note the significant drop in inflammation in groups treated with metformin, rapamycin and probiotics (group 4B and 8B) where the lowest scores were obtained (0.1). The values represent mean ± SEM (n = 6). Significance of p<0.05 was indicated by (^*^) when compared to diabetic control, and non-diabetic control (^**^); and (^***^) indicated significance between G8A and 8B.

The microscopic findings in the descending colon were scored according to the aforementioned criteria by 2 different observers (Figures 6, 7). Concerning the descending colon, most of the alterations were recorded in groups 1 and 5, not treated controls, non-diabetic and diabetic, (Figure 6A-6F), respectively.

In group 1, there was a marked hyperplasia with a major loss of goblet cells (black circles), polyp formation, and inflammatory cells infiltration (black arrows). The average score of histopathological changes in G1 was 2.4 out of 3. Similar but relatively more severe alterations were encountered in group 5, the diabetic mice with a score of 2.6. Treatment with rapamycin decreased the alterations in G2 and G6 with a score of 1.3 in both based on the presence of less inflammatory cell aggregates (black arrows), less disruption in mucosal architecture and irregularities in the epithelial lining as well as submucosal edema (star). Similarly, treatment with metformin in G3 and G7 improved the alterations seen in G1 and G5 with more improvement in G3 the non-diabetic (score of 0.9) compared to diabetics (score of 1.3). However, the inflammatory reaction was more persistent in G1 (black arrow). On the other hand, the combination (Met+ rapamycin) did show more decrease in the morphological alterations especially in the non-diabetics G4A (score 0.6) compared to G8A (score of 1.0), close to normal with little submucosal edema and inflammatory cells.

Concerning the use of probiotics plus the combination in G4B and G8B, the tissues of the colon were almost normal with scores of 0.1 both in G4B and G8B.

In brief, there was amelioration to various degrees in the colonic tissues with more effect in the presence of the combination therapy with or without probiotics; on the other hand, the histology was close to normal in presence of probiotics (Figures 6 and 7).

#### Mast cells number variations

Concerning mast cells, they are normally present in intestinal tissues, they are activated during inflammatory reaction; they degranulate and increase in number. One of the features of inflammatory bowel diseases is mast cell stimulation, secretion and hyperplasia. Hereby, the study of the colonic tissues stained with toluidine blue showed that the high scores encountered in G1 (12.2) and G5 (11.7) decreased. Moreover, the mast cell number decreased with the administration of metformin alone and rapamycin alone in a significant way when compared to controls (p<0.05); G2 (5.8) and G6 (5.6) for rapamycin, while G3 (3.4) and G7 (4.2) for metformin. The greatest decrease was obtained with the combination of metformin and rapamycin with probiotics; G4B (0.9) and G8B (0.8) with a significant reduction of 92.6% and 93.2% respectively (p<0.05). However, in the diabetic groups G4A and G8A, there were no additive effects of metformin and rapamycin 3.4 and 4.2, respectively (Figure 9).

**Figure 8:**
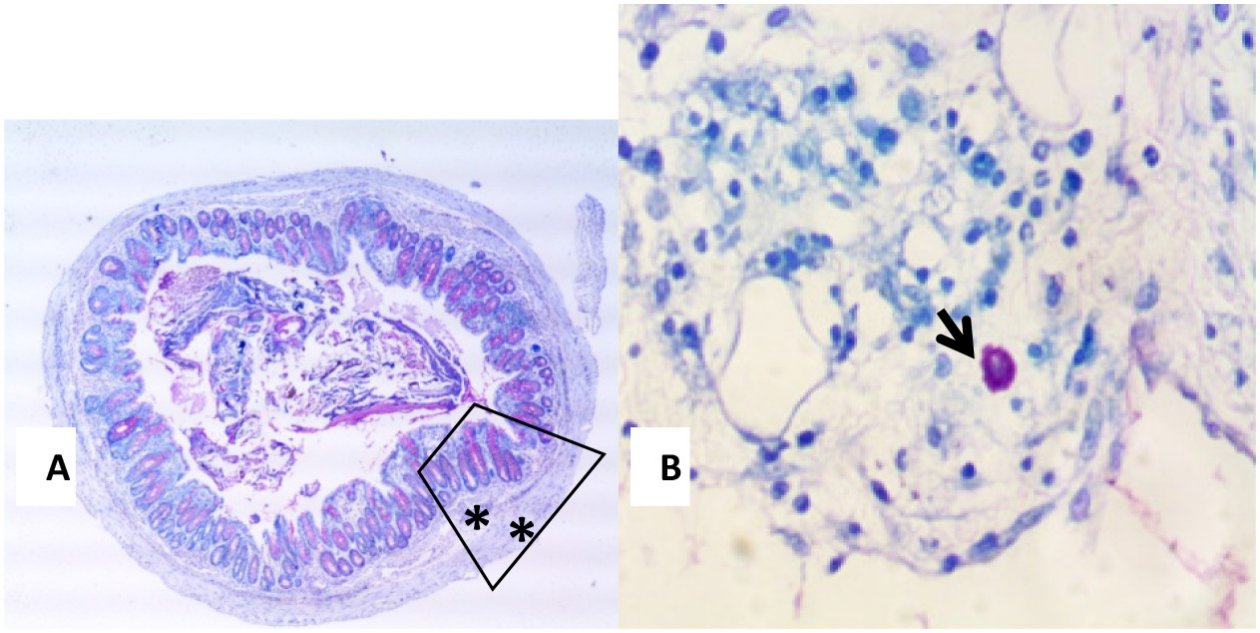
**(A** and **B)** Toluidine Blue stained colonic section (13A) showing a typical mast cell (black arrow) seen in the submucosa of an inflamed colon (13B 400X magnification).

**Figure 9:**
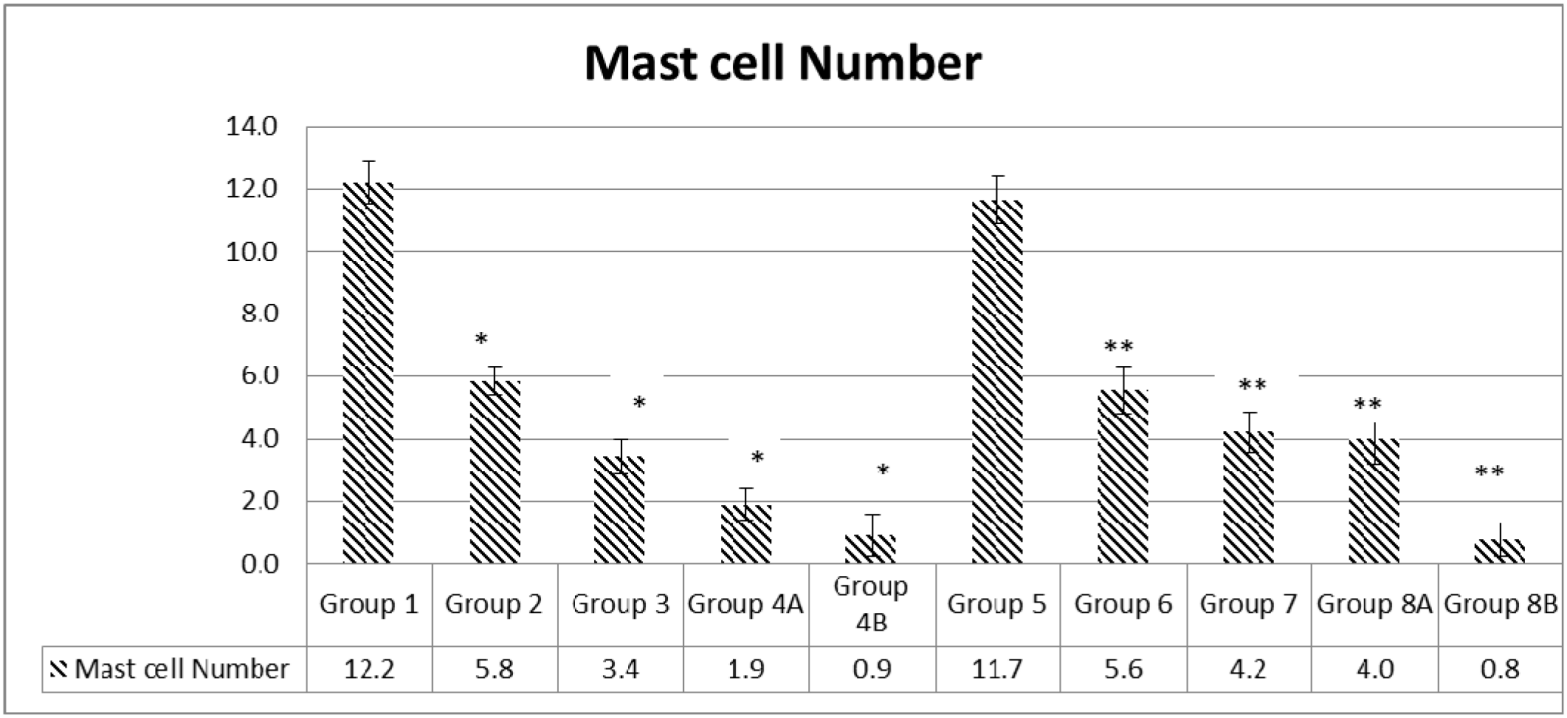
Quantification of mast cell numbers Note that the highest numbers of mast cells were obtained in groups 1 and 5. Treatment with metformin and rapamycin alone or in combination with probiotics were able to reduce the mast cells number in a significant manner. The lowest values were obtained in group 4B and 8B when probiotics were administrated to mice in addition to the metformin and rapamycin’s combination. The values represent mean ± SEM (n = 6). Significance of p<0.05 was indicated by (^*^) when compared to diabetic control, and non-diabetic control (^**^).

### Reactive oxygen species changes

Besides, the inhibition of reactive oxygen species (ROS) was significant. In general, cancer cells increase their rate of ROS production compared with normal cells. In this experiment, ROS were assessed in all the colonic samples of the various groups using the DHE staining technique.

In non-diabetics: groups 2, 3, 4A and 4B, the different treatments were able to reduce ROS production in a significant manner when compared to controls in G1 (P = <0.001). In addition, a similar pattern was noted in diabetics: groups 6, 7, 8A and 8B compared to control G5 ROS reduction was significant (p< 0.05). In both G1 and G5, the ROS values were similar and relatively very high (7268 units), regardless of the diabetic or non-diabetic status of the mice.

Actually, in non-diabetics, rapamycin decreased significantly ROS production from 7268 in G1 till 1923 in G2, and a similar trend but to a lesser and also significant degree in diabetics (3173 units). As for metformin, grossly the effects were similar (G3=1695 and G7=2150), significantly less than G1 and G5, respectively, p<0.05.

**Figure 10:**
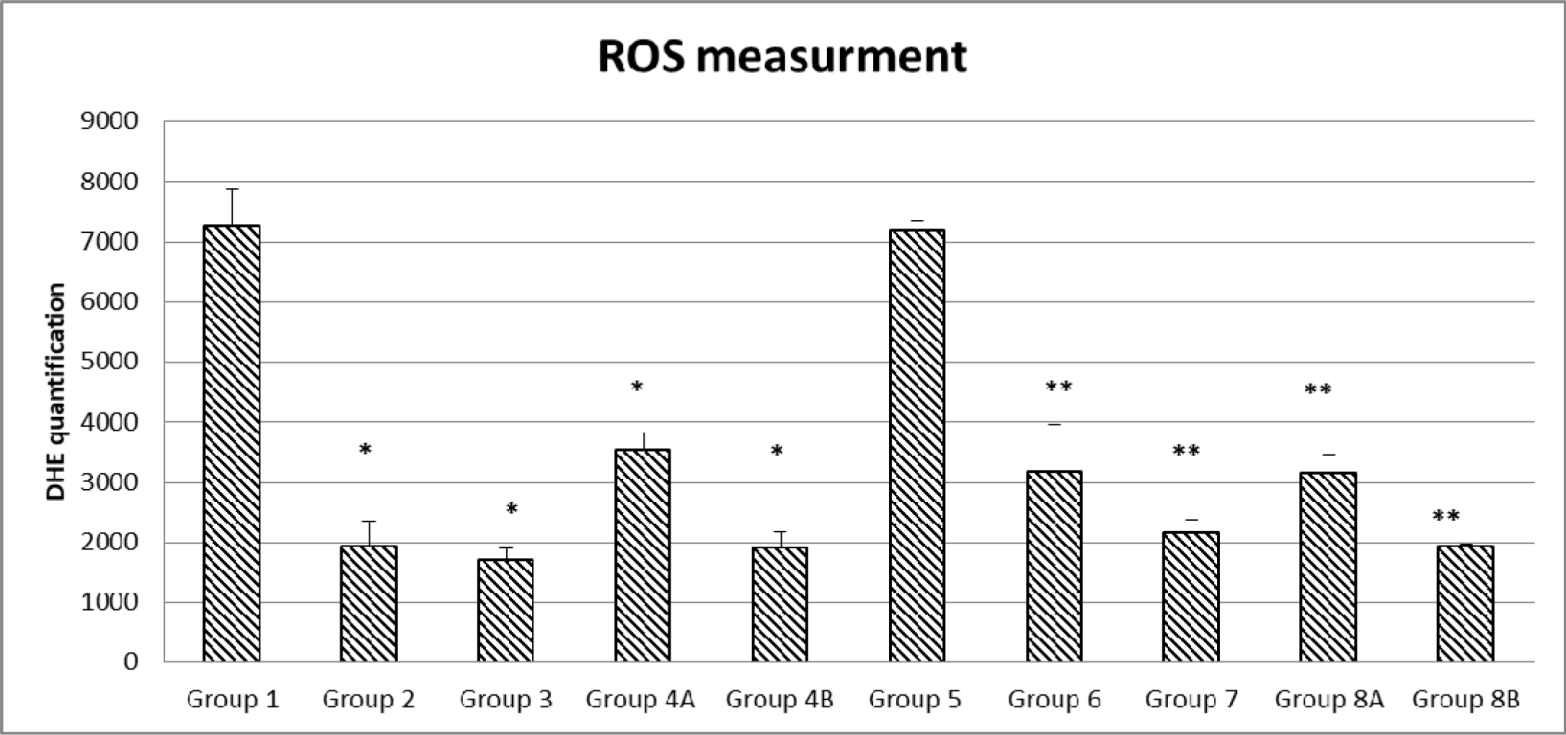
Quantification of ROS formation in the different diabetic and non-diabetic groups Note that the highest ROS levels were obtained in the non-treated groups (1 and 5), the different treatments and their combinations were able to reduce ROS levels to a various extent in a significant manner. The values represent mean ± SEM (n = 6). Significance of p<0.05 was indicated by (^*^) when compared to diabetic control, and non-diabetic control (^**^).

**Figure 11:**
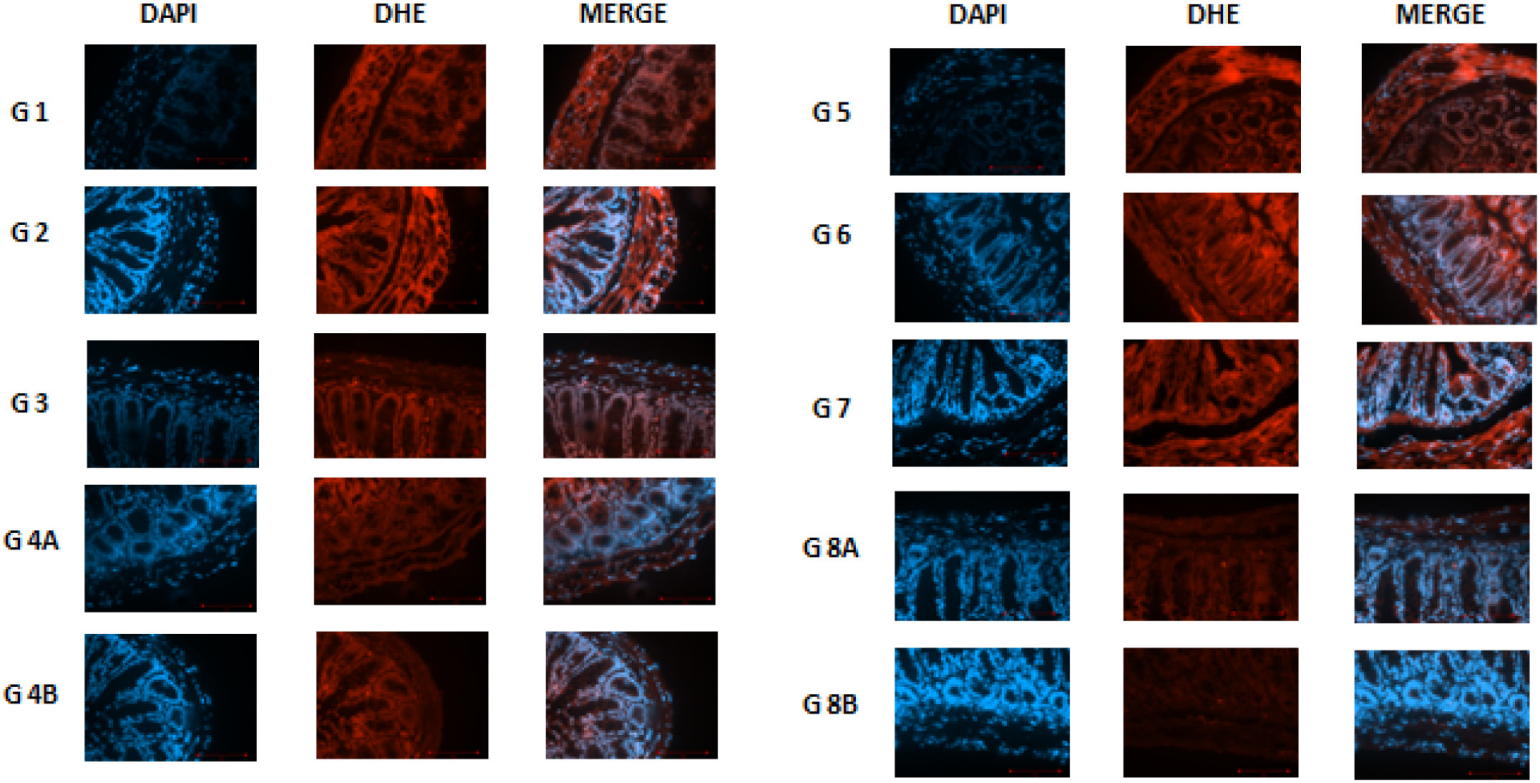
DHE staining in non–diabetic animals, groups 1, 2, 3, 4A and 4B, as well as in diabetic animals (groups 5, 6, 7, 8A and 8B) showing the difference in stain intensity when comparing the non-treated group 1 and 5 to the treated groups Note that the lowest red fluorescence was obtained in group 4B and 8B treated with the combination of metformin, rapamycin and probiotics.

On the other hand, there was no additive effect for the metformin and rapamycin combination, the ROS values were significantly less than the non-treated G1 and G5 but relatively more than either metformin alone or rapamycin alone (G4A=3533 and G8A=3147). Furthermore, the presence of the probiotics in the combination therapy made a significant difference in both diabetics (G8B=1903) and non-diabetics (G4A=1918). In brief, all treatments significantly decreased ROS production to various extents; however, the lowest values were with metformin and the combination with probiotics with the absence of additive effect between metformin and rapamycin. All of the differences in the mean values of ROS production among the treatment groups are greater than would be expected by chance; there exist a statistically significant difference (Figures 10 and 11).

### Molecular analysis of relevant genes and proteins

#### Gene Expression of AMPK, mTORC and KI67

Repeatedly, AMPK gene expression was close in most non-diabetic groups, close to 1.0 in G1, G2 and G3 and decreased by about 31% in G4A (=0.69) and 39% in group G4B (0.63), with combination therapy or combination plus probiotics, respectively.

**Figure 12:**
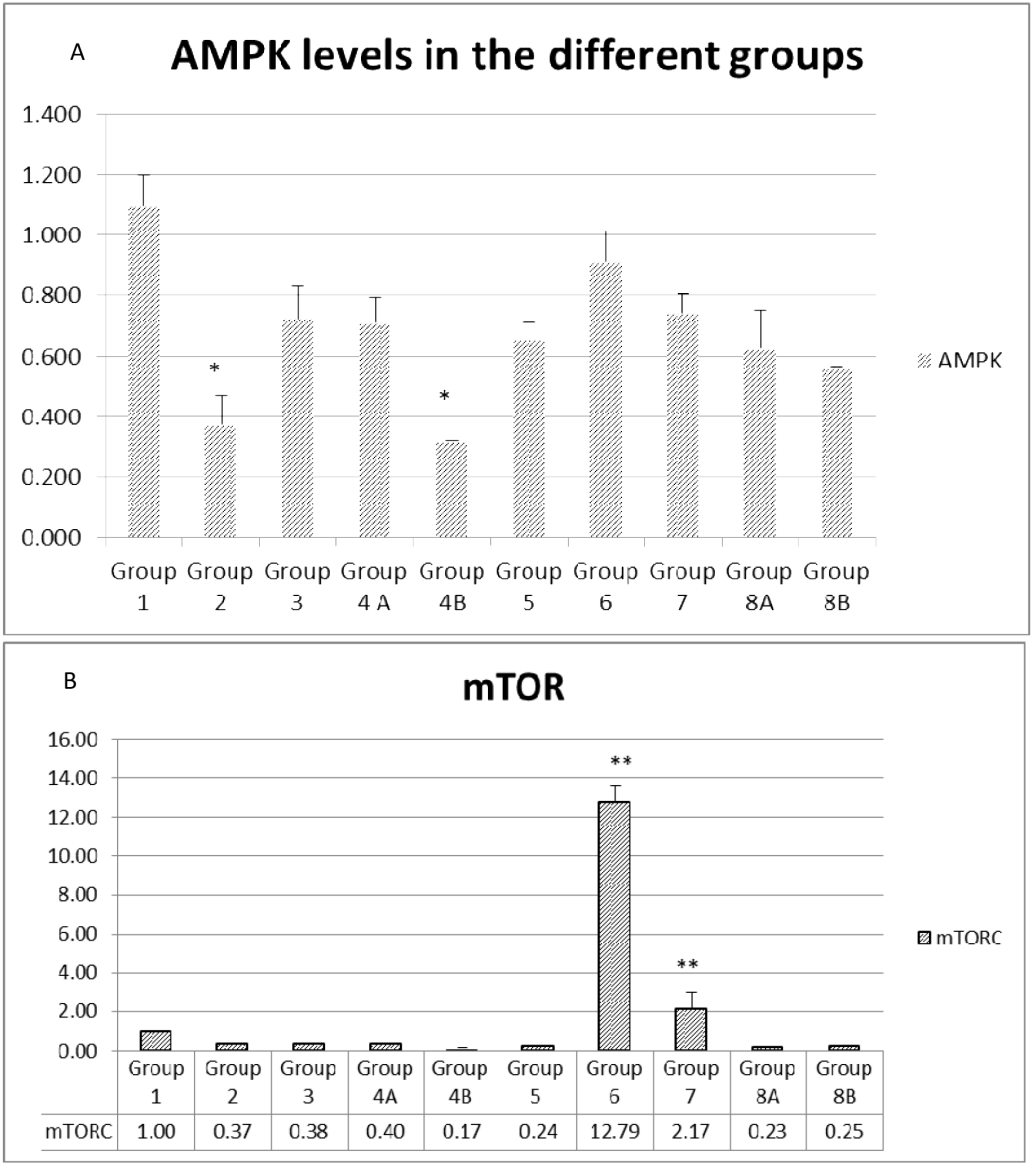
**(A** and **B)** Expression of main genes involved in colorectal carcinogenesis. The values represent mean ± SEM (n = 6). Significance of p<0.05 was indicated by (^*^) and (^**^) when compared to diabetic control and non-diabetic control respectively.

On the other hand, the non-treated diabetic mice in G5 expressed less AMPK by about 31% than the non-diabetics in G1. In addition, the expression in the rest of the diabetics G6, G7 and G8A was not significant. it was less then G1 G2 and G3. In diabetics also, a slight increase in AMPK was observed when metformin and rapamycin were administered alone or in combination. However, when probiotics were added to the combination, a decrease of about 20% was observed in group 8B compared to 8A, similar to non-diabetics with combination G4A and close to 4B. It seems that the triple treatment could show a distinct difference compared to the other groups (Figure 12A).

Concerning mTORC expression, it was suppressed in all groups except in the rapamycin treated diabetics, where the value was highly significant in G6=12.79 compared to very low expression in all other groups. In brief, only rapamycin treatment upregulated mTORC (Figure 12B).

**Figure 13:**
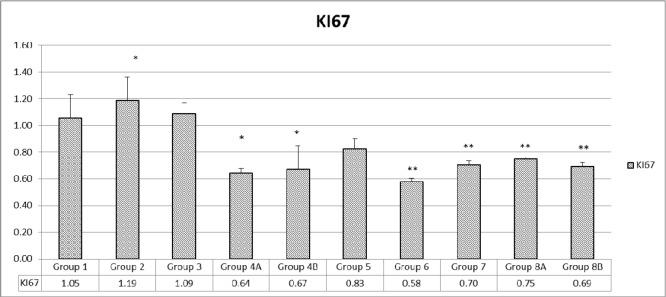
Assessment of proliferation via KI67 The values represent mean ± SEM (n = 6). Significance of p<0.05 was indicated by (^*^) and (^**^) when compared to diabetic control and non-diabetic control respectively.

As for KI67 genes, whose level of expression indicates the proliferation of the cells, the data profile was close to the AMPK expression profile. Partial inhibition of proliferation was encountered when combination treatment was used in non-diabetic with (33%) or without (36%) probiotics (Figure 13).

Some decrease of proliferation was encountered in all diabetics: 20% in G5, 42% in G6 with rapamycin treatment, 30% with metformin treatment in G7, 25.0% with rapamycin and metformin G8A and 31% when probiotics were added to the combination. Therefore, the combination, with or without probiotics decreased proliferation by 20-40% (Figure 13).

#### Gene expression of pro-inflammatory cytokines: IL-3, IL-6 and TNFα

**Figure 14:**
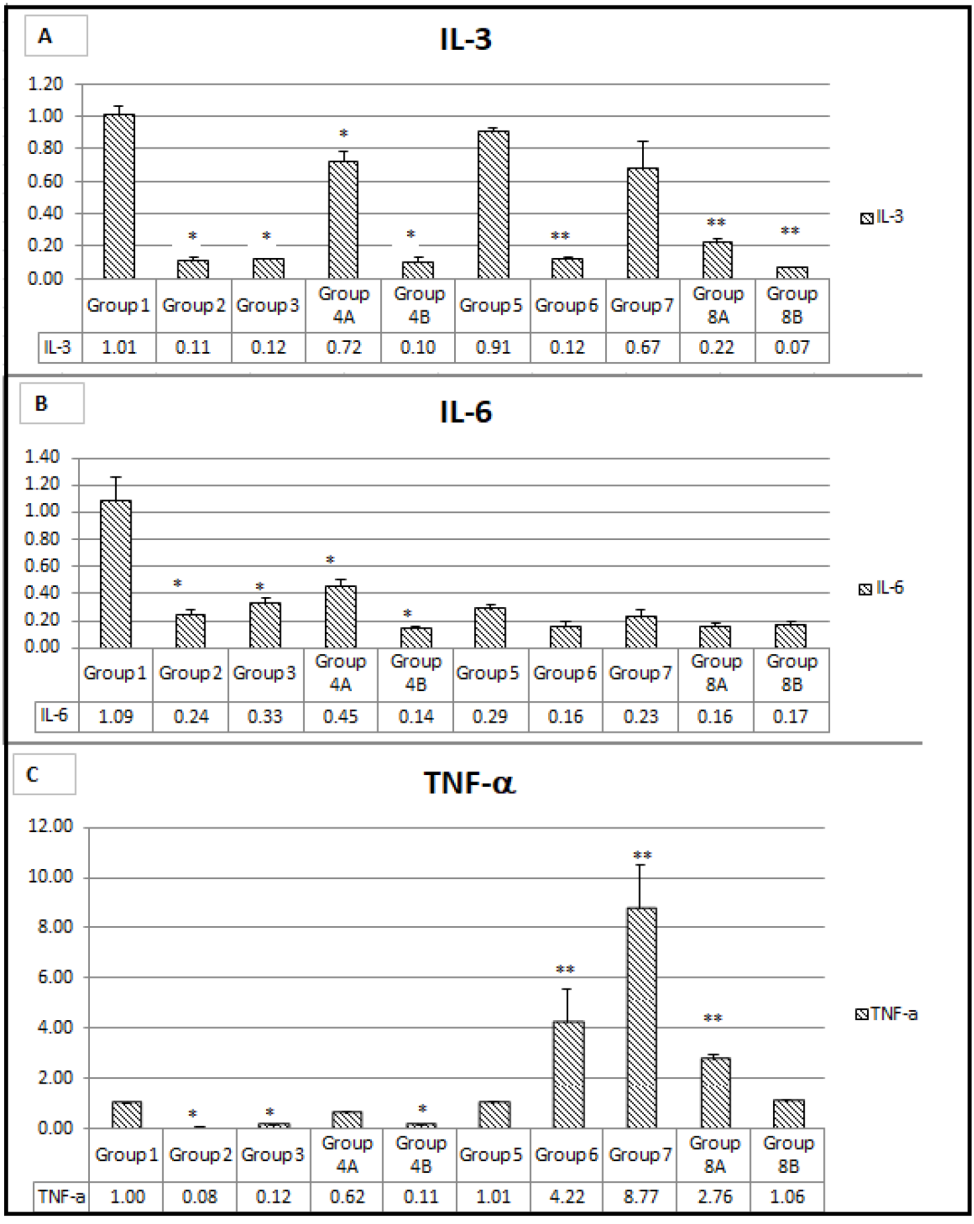
**(A, B, C)** Expression of main genes involved in inflammation. The values represent mean ± SEM (n = 6). Significance of p<0.05 was indicated by (^*^) and (^**^) when compared to diabetic control and non-diabetic control respectively.

As expected the expression of Il-3 genes was relatively the highest in the non-treated mice (G1=1 and G5=0.9). It was inhibited significantly in non-diabetics by rapamycin alone and metformin alone by 89% and 88% respectively. Inhibition by the combination therapy plus probiotics was by 90%. However, again the combination of metformin and rapamycin together inhibited by about 78% in diabetics; again, there were no added effect but rather may be a competitive effect of the 2 drugs. On the other hand, in diabetics, the inhibition was very significant in all groups (G6=90%) G8A=80%, and G8B=90%, However, metformin alone inhibited the expression by only 33% compared to G5 which expressed IL-3 by 91% (Figure 14A).

Concerning the other interleukin IL-6, its gene expression was suppressed in almost all groups to various extents except in G1, the non-diabetic, non-treated group of mice.

The combination plus probiotics G4B and G8B had relatively the highest suppression, 86% and 83%, respectively. However, the combination without probiotics and rapamycin alone had 84% suppression and 76% in non-diabetics. In brief, there was an additive effect of both drugs in the diabetics but not in the non-diabetics (Figure 14B). In the non-treated diabetic mice, the expression of IL-6 was low, about 24% with rapamycin, 33% with metformin and 45% with the combination, and in all the rest of the groups the IL-6 expression was less, G2=24% G3=33% G4B=45% G5=30% G6=17% G8A and G8B=17%. However, in non-diabetics, met and rapamycin did not have an additive effect but rather a competitive effect G4A=45% (Figure 14B).

As for TNFα, its gene expression was extremely inhibited >90% in all the non-diabetic groups, and even in the non-treated mice both diabetics and no-diabetics. However, the TNFα gene expression was relatively elevated 4.ww times compared with rapamycin treatment and 8.77 times with metformin treatment compared to G1 (1.0) and G5 (1.01). There was no additive effect, however, in the presence of probiotics G4b and G8B the inhibition was almost complete (Figure 14C).

In brief, the administration of metformin alone or rapamycin alone induced a decrease in the expression of the inflammatory markers, IL-3, IL-6 and TNF-ɑ in both diabetics and non-diabetics. The lowest scores were obtained in diabetic and non-diabetic groups taking the triple-therapy (metformin, rapamycin and probiotics) when compared to non-treated controls. However, a slight increase in IL-3, IL-6 and TNF-α was noted when combining rapamycin and metformin shedding light on possible alternative signaling pathways (Figure 14A, 14B, 14C).

The three inflammatory markers studied had the similar expression profile to a great extent, implicating that all treatments produced a prominent decrease in the inflammatory response which forms a favorable environment for colorectal carcinogenesis development and progress.

#### Expression of mTOR and p-mTOR at protein level in tumors

**Figure 15:**
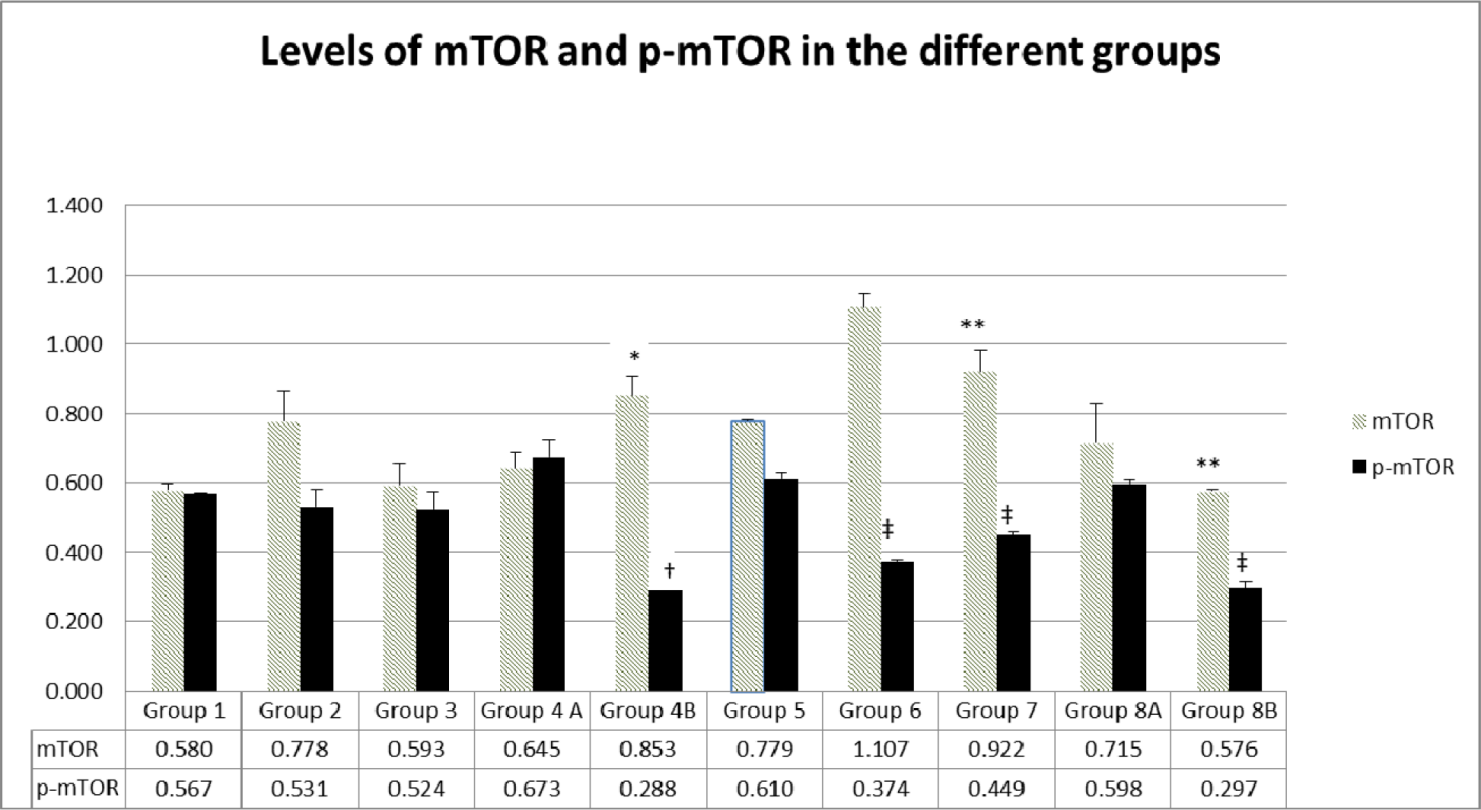
Expression of mTOR and p-mTOR at protein level in the different groups The values represent mean ± SEM (n = 6). Significance of p<0.05 was indicated by (^*^) and (†) when compared to diabetic control, (^**^) and (‡) when compared with non-diabetic control (^**^) for mTOR and p-mTOR respectively.

Data emanating from western blots performed on proteins extracted from tumor sections and assessing the effect of the different treatments on the expression of mTOR and its phosphorylated form p-mTOR, showed different levels of inhibition in the diabetic and non-diabetic animals (Figure 15).

Data showed that the treatment with rapamycin increased the levels of mTOR in both diabetics (G6 compared to G5) and non-diabetics (G2 compared to G1). On the other hand, treatment with metformin introduced no change in m-TOR and p-mTOR (G3 versus G1) in non-diabetics but a significant increase in diabetics (G7 compared to G5). Using the combination of metformin and rapamycin, did not introduce any significant variations, thus leading us to conclude one more time that the 2 drugs do not have an additive effect on mTOR. However, by adding probiotics to the combination, mTOR expression increased in non-diabetics (G4B=0.85 compared to G1=0.58) and decreased slightly in diabetics, G8B=0.57 compared to G5 0.77).

The p-mTOR decrease was really significant when probiotics were added to metformin and rapamycin in both non-diabetics and diabetics. In addition, p-mTOR was also significantly inhibited with either rapamycin or metformin treatment in diabetics (G6=0.37, G7=0.449 vs G5=0.61).

In brief, it is important to note that the treatment with rapamycin alone or metformin alone was able to inhibit mTOR activity *via* decreasing its phosphorylation. However, the effect or rapamycin was more significant. The highest inhibition of p-mTOR was obtained when adding probiotics to the combination in diabetic and non-diabetic mice. In addition, there was no additive inhibitory effect of metformin and rapamycin, but the opposite is true, a slight increase in p-mTOR was noted (Figure 15).

## DISCUSSION

Clinical observations and studies indicate that the prevalence of diabetes in newly diagnosed cancer patients ranges from 8 to 18%, suggesting bidirectional association between these 2 diseases [26–28]. In addition, publications in the past 5 years have also suggested the link between first line hypoglycemic medications like metformin and the delay in initiation of cancer [29–31]. However, the mechanism is still unclear, despite the fact that metformin is capable of activating AMPK involved in tumorigenesis [32].

On the other hand, rapamycin, originally used as an antifungal agent [33], it was later approved as a potential anticancer drug [34], a specific inhibitor of the mammalian target of rapamycin (mTOR) signaling pathway, master regulator of cell growth and metabolism implicated in a number of diseases including diabetes and cancer [35]. However, the modest effect of rapamycin-based therapy has prompted investigators, including our laboratory, to consider combination therapy of metformin and rapamycin, especially that metformin administration significantly reduced CRC incidence [36].

Such *in vivo* data also supported by *in vitro* data in our laboratory, using either metformin alone, rapamycin alone or a combination of the 2 drugs, on decreasing the proliferative activity of HCT116 and HT29 colonic cell lines, whereby a physiological and supraphysiological doses the 2 drugs were not toxic and well tolerated by the animals. In this study, despite the fact that rapamycin and metformin were used in combination, with or without probiotics, they did not lead to unfavorable toxicity, as depicted by histological assessment of liver and kidney biopsies, while triggering tumor regression, thus suggesting their potential value for treatment of CRC in diabetes.

As expected, the clinical profile of the controls untreated xenografts was the worst. The individual treatments helped removing some of the symptoms and signs but more so was achieved with the combination therapy, in particular, when probiotics were added to the combination. Such an effect was more remarkable in diabetics versus non-diabetics. Based on clinical observations, the highest disease activity (DAI) was detected in the non-treated animals to significant extents; however, each drug had some positive effect on the DAI, more so for metformin than rapamycin. On the other hand, there were no significant additive effects between metformin and rapamycin except when supplemented with probiotics, then all symptoms and signs decreased significantly in all diabetics and non-diabetics.

In line with published reports, metformin significantly reduced or delayed the occurrence of CRC development in the animals; it worked as an independent protective factor against CRC [36]. It might have exerted its cancer chemo preventive effects by suppressing the transformation and hyper proliferative processes that initiate carcinogenesis [37]. Similarly, rapamycin has also exercised its potential as an anticancer drug in this rapamycin-sensitive cancer model, but to a very low level, compared to metformin alone or the combination plus probiotics. In brief, the 2 drugs showed remarkable effects in preventing or slowing down the progress of the development of the xenograft. Such an effect was consistent in delaying tumorigenesis particularly when probiotics were added in G4B (for 3/5) for 14 days and in G8B (3/5) for 15 days; p-mTOR has been suppressed significantly in both groups.

At the same time, there was a decrease in size of the tumor in almost all treated groups, to various extents, compared to untreated animals. Again, little but significant effects were encountered with each drug separately with no indication of added effects except when probiotics were in the combination. The probiotics treated animals had decreased size and frequency of all tumors in both diabetics and to a lesser extent in non-diabetics (Figures 7 and 8). Such effects were very significantly correlated with improvements in pathological alterations in the various groups. Each of the 2 drugs had moderate effects which did not add, but were conspicuous in terms of smaller size, lower cell density and less inflammatory cells leading to less production of pro-inflammatory agents IL-3, IL-6 and TNF-α and less ROS production.

Concerning the colon, most alterations in colonic tissues improved remarkably by treatment with either drugs. However, such improvements were more evident when combination therapy was adopted in G4A (0.6/3) and G8A diabetics (1.0/3), and even more prominent when probiotics were added, reaching a histological status very close to normal. The inhibition of mast cells followed a similar pattern and the panoply of inflammatory mediators, secreted by these cells, decreased as the number of mast cells decreased to various extents in the different groups commensurate with decrease in pro-inflammatory mediators and the improvement or limitation of the inflammatory reaction in the various groups. Again, no signs of added effects among the 2 drugs, but the presence of probiotics, one more time, did make a positive impact on the tissues and cells of the colon. Were the drugs working by different mechanisms, and how did the probiotics make the difference? Did they affect ROS production and controlled ROS activity?

In general, cancer cells increase their rate of ROS production, compared to normal cells, and increase their susceptibility to ROS-manipulation therapies. The association of ROS with cancer cells could be oncogenic at high levels [38] thus promoting cancer cell proliferation, survival, angiogenesis and metastasis [39]. To maintain redox balance, cancer cells increase their antioxidant capacity by scavenging excess ROS. Data emanating from this study showed that the 2 drugs rapamycin and metformin could decrease significantly ROS levels in both diabetics and non-diabetics without showing any added value to the combination therapy even with probiotics. As expected, the highest values of ROS were associated with the non-treated xenografted animals with or without diabetes. The mechanism of ROS reduction by the 2 drugs is probably independent of that of probiotics. This increase in oxidative stress induced more cancer cell death and smaller tumors [40]. The cancer cells were as sensitive in both metformin and rapamycin treated animals. Actually, ROS was probably maintained in the various groups at a level that allows for the activation of protumorigenic signaling pathways. Hence, strategies to eliminate ROS or produce ROS may be effective in cancer therapies. In this study, both drugs led to a remarkable decrease in ROS production and consequently a reduction in its pro-cancer effect. ROS could have also oxidized and inactivated MAPK phosphatases and MAPK/ERK pro-proliferative signaling [41]. They could have also promoted tumor cell survival through the activation of NFKB and NRF2 transcription factors that upregulate the expression of antioxidants to evade ROS mediated cancer cell death [42].

Moreover, data showed that high values of ROS promoted tumor angiogenesis and metastasis as detected in G1 and G5 control non-treated groups. Such cases are usually associated with poor prognosis and activate the AMPK [43]. Actually, ROS levels increased in these solid tumors and AMPK was activated to probably promote NADPH production. On the other hand, loss or significant decrease of AMPK by metformin or rapamycin, could have prevented oncogenic transformation [44]. In brief, ROS has been shown to regulate numerous signaling pathways (e.g. MAPK PI3K/Akt and JNK pathways) and decreasing ROS levels could prevent cancer cell proliferation. Therefore, developing methods to decrease intracellular ROS levels and prevent cancer cell proliferation is an attractive field. So far, ROS manipulation strategies have previously focused on antioxidant therapy. A better understanding of the molecular mechanisms of ROS signaling in cancer and the identification of specific ROS targets may provide novel therapeutic avenues for treating cancer. Could probiotics be performing this task? Data in this study do not clearly support this claim.

On the other hand, emerging research on CRC points out to a complex network of genetic alterations leading to dysregulation of multiple pathways. Moreover, as proposed by CRC subtyping consortium, there are 4 major molecularly distinguishable subtypes of CRC [45] and when coupled with T2DM, they tend to have a less favorable prognosis [46]. Further, patients with inadequate glycemic control may have an even higher risk of CRC and need to receive polytherapy [47]; a potential indication for metformin and rapamycin. In this context, observational studies have suggested that some anti-hyperglycemic agents like metformin, could decrease or prevent cancer risk [48] [49] could activate AMPK, a central regulator and an important target for controlling human diseases including T2DM and cancer. AMPK could cause cell cycle arrest in response to metabolic stress through a number of mechanisms [50]. In addition, AMPK might protect sometimes tumor cells against action of cytotoxic agents and hypoxia once tumor is established, or even delay the onset of tumorigenesis [32]. Along this line, studies on the AMPK have shown that mTORC1 and RNA polymerase I transcription factor TIF-1A, both of which are required for rapidly proliferating cells, are under the control of AMPK [32].

In this study, AMPK levels in non-treated diabetics (G5) were 31% lower than in non-treated non-diabetics (G1). When metformin was administered alone, a slight decrease in AMPK was also observed. Notably, when probiotics were added, these levels decreased remarkably in diabetics and non-diabetics by 40%. This behavior remains unexplained and requires further explanation as to decipher the mechanism which lowered AMPK despite the administration of metformin, an AMPK activator.

On the other hand, the mTOR pathway components are over expressed in CRC [51]. The mTOR combines with raptor(regulatory associated protein of mTOR) to constitute mTOR complex 1(mTORC1) and rictor (rapamycin-insensitive companion of mTOR) to make mTORC2 [52].Consequently, mTOR also emerged as a compelling molecular target for treating several malignancies [53]. There are two different types of mTOR inhibitors, (1) ATP competitive mTOR inhibitors that block the activity of mTORC1/mTORC2, and(2) rapamycin analogs that influence the activity of mTORC1 [54]. Which one applies in this study?

Actually, rapamycin inhibits the mTORC1 activity, suppresses the proliferation of the adenoma cells, inhibits of tumor angiogenesis and decreases the size and number of polyps [55]. It also inhibits tumor growth in a dose dependent reduction in HCT116 xenografts [56]. All such effects are encountered in this study. The mTORC gene expression was minimal or not expressed at all except in the rapamycin treated diabetic animals where it exhibited relatively high values. However, the expression at the protein level was relatively highest in the rapamycin treated diabetics in concordance to the gene expression level. On the other hand, the expression of p-mTOR protein was relatively more suppressed in the combination therapy especially when probiotics were added, both with diabetics and non-diabetics. Among the rest of the groups mTOR and p-mTOR were moderately expressed. Consequently, the clinical improvements of the health status of the animals and of the cancers did not seem to be related to the p-mTOR levels of suppression.

Data of K167 depicted that the proliferation of the cancer cells continued in these suppressed groups but at a much lower pace, a phenomenon which might explain the decrease in size of the tumors in the suppressed groups. Such changes were in concordance with the lower levels of gene expression of molecules involved in the mTOR pathway. The uncontrolled mTORC1 mediated signaling could be basically explained by the intricate signaling network of mTOR and possibly the inability of rapamycin to completely block mTORC1 mediated signaling events which could be explained by the presence of several feedback loops, and the upregulation of compensatory pathways that promote cell survival and growth.

As for the proinflammatory cytokines, IL-6, IL-3 and TNF-α gene expression was remarkably suppressed in all groups, to various extents, except in the non-diabetic non-treated animals. There was obviously no added effect of the 2 drugs except in the presence of probiotics. In brief, the drugs did control to various extents the inflammatory process. However, the inflammatory cytokines which were supposed to activate mTOR [57] were suppressed, in particular, when using the combination of rapamycin and metformin, in presence or absence of probiotics. It seems, in this case that the treatment with rapamycin might be further potentiated with the antidiabetic drug metformin [58, 59] and even more so by probiotics. Such results were more evident when the p-mTOR protein was assessed; it depicted a much lower expression than the mTOR especially with metformin, rapamycin, and the 2 drugs in presence of probiotics, both in diabetic and non-diabetic animals. The additive effect of both drugs was not significant. Such results are congruent with the other data collected on proliferation, ROS production, mast cells decrease, inhibition of colonic inflammation, improvement of histological alterations, lower DAI severity and, to some extent, with the decrease in tumor volume in both diabetics and non-diabetics. The presence of probiotics in the combination of metformin and rapamycin led through one or more mechanisms, to the suppression of tumor size, delay in their development, significant inhibition of the inflammatory reaction, as well as a decrease in ROS production, lower cell proliferation, significant decrease in AMPK and inhibition of the phosphorylated mTOR. Further experiments are needed in this area to elucidate the complexity of the pathways involved and eventually the specific targeted molecules as well as the exact role of probiotics and their mechanism of action.

**Figure 16:**
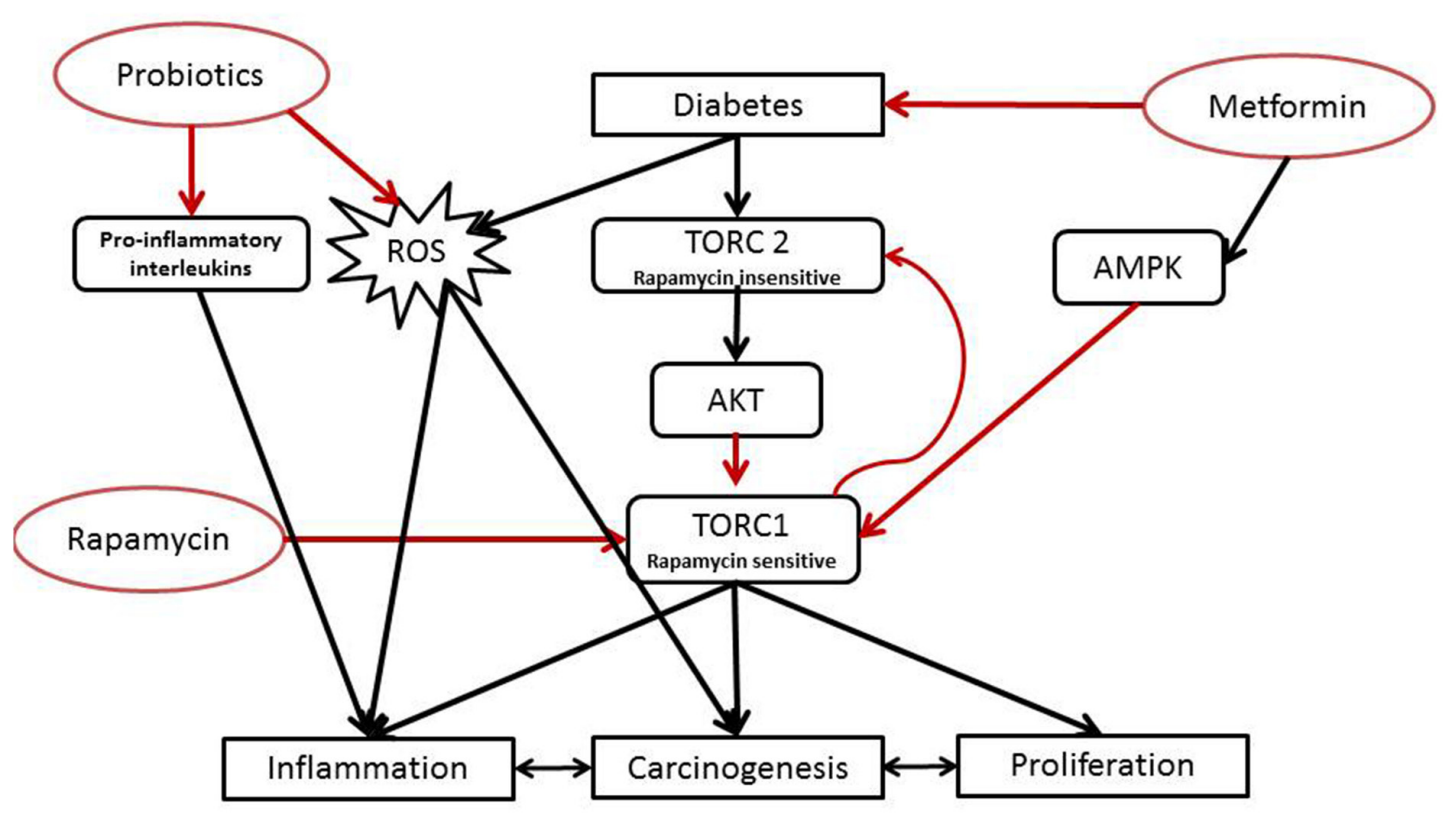
Proposed mechanism of action of the tri-therapy

The emergence of combination therapy with rapamycin and metformin and/or probiotics may further increase efficacy and bypass possible feedback activation of survival pathways. Significant promise remains for the discovery of new specific signaling inhibitors to reduce mTORC activation, in monotherapy or in polytherapy and decipher the place and role of probiotics in this complex process (Figure 16).

The findings reported in this article suggest that modulation of the gut microbiome with probiotics in combination with the anti-proliferative agents, i.e. rapamycin and the antidiabetic drug metformin, a potential prebiotic agent, could constitute or be a part of a new preventive and or therapeutic strategy for CRC management, one of the most common cancers worldwide.

In conclusion, the synergetic action of rapamycin and metformin in association with the probiotics led to reduced expression of early lesions in CRC such as aberrant crypt foci (ACF). Supplementation of the combination by probiotics for two weeks, in mice with xenografts could possibly lead to a decrease in the formation of classical ACF, an increase in apoptosis and reduced rates of inflammation, PCNA and *p* 53 positive cells.

It is likely that the established chronic inflammatory process combined with dysbiosis could contribute to an oxidative stress, an increase in reactive oxygen and nitrogen species, as well as pro-inflammatory cytokines; key factors involved in the development of CRC. Such factors were reduced by the various treatments to different extents. To reduce or inhibit carcinogenesis linked to oxidative stress, a strategy of chemo prevention involving the administration of exogenous compounds which intervene with the proliferation of cancer cells and with the blocking of their oncogenic transformation, as well as lowering hyperglycemia, could probably constitute a novel strategy. Would metformin and rapamycin coupled with probiotics serve the purpose?

## MATERIALS AND METHODS

### Animals

Fifty male NOD/SCIDs mice 6-8 weeks old, weighing 25–30 g, were housed in Individually Ventilated Cages (IVC) at the transgenic unit of the Animal Care Facility of the American University of Beirut, in a controlled temperature (21°C±2°C) and humidity, with an alternating 12-hour light/dark cycle. Standard Laboratory pellet formula and tap water were provided ad libitum. All animal treatments adhered strictly to institutional and international ethical guidelines of the care and use of laboratory animals. The experimental protocol was approved by the Institutional Animal Care and Use Committee, American University of Beirut, Lebanon (identification number # F16-00328).

### Experimental design

The animals were divided into 2 main groups, (1) diabetic and (2) non-diabetic. They were all subject to one subcutaneous injection of 3×10^6^ HCT116 cells suspended in 200 μl normal physiological saline, in the flank which produced xenograft tumors after 9 days.

Diabetes was induced using Streptozotocin (STZ) (S0130-50MG-Sigma Aldrich), a N-nitroso-containing compound that acts as a nitric oxide donor in the pancreatic islets of Langerhans; induces death of insulin-secreting cells, and thus producing an animal model of diabetes. Two Streptozotocin intra-peritoneal injections at day 1 and 8 were able to induce diabetes (glycemia >150 mg/dl).

For metformin (Glucophage) treatment, it was dissolved in drinking water to attain the dosage of 150 mg/kg body weight. The water was changed daily and measured for water intake. Metformin daily treatment was initiated 7 days before inoculation of the tumor cells and was continued until sacrifice.

As for rapamycin, (37094-10MG-Sigma Aldrich), it was stored at -20° C; diluted with DMSO and administered via 100 μl i.p injections (3 injections per week) at a dose of 0.5 mg/kg. The first injection of rapamycin was administered to the respective groups, 1 week after the onset of tumors, *i.e*. when tumor size reached 50mm^3^.

Probiotics (Probiolife®), a symbiotic mixture, combining the most studied strains of probiotics such as lactobacillus rhamnosus, Saccharomyces boulardii, Bifidobacterium breve, bifidobacterium lactis, lactobacillus acidophilus, lactobacillus plantarum, lactobacillus reuteri, in addition to prebiotics and zinc, were administered to mice in their drinking water 2 weeks before sacrifice. One capsule was dissolved in 1.75 L of autoclaved tap water with a concentration of 10^8^ CFU/ml. Fresh solution was given to the animals every 2 days.

Each of the 2 main groups of 25 mice was subdivided into two subgroups, 15 mice treated with metformin, 150 mg/kg body weight administered in drinking water, and 10 mice not treated. A total of 10 groups of 5 animals each were reached: group 1 received no treatment and was considered as control; group 2 received rapamycin only; group 3 was treated with metformin alone; group 4A was treated with both metformin and rapamycin; and group 4B received probiotics in addition to metformin and rapamycin. On the other hand, the diabetic mice, treated with STZ were divided similarly: group 5 received nothing; group 6 rapamycin; group 7 metformin; group 8A metformin and rapamycin; and lastly group 8B received probiotics in addition to metformin and rapamycin.

### Monitoring

Mice were monitored for glycemia pre and post STZ injections and weekly afterwards. Weight changes (weight loss), stool aspect (loose or bloody), fur shape and activity were daily checked. In order to determine tumor volume, the maximal longitudinal (length) and transverse diameters (width) were measured using a caliper square, once per week. Tumor volumes were calculated using the formula: *tumor volume=length×width×width/2.* The scores were recorded to calculate disease activity index (DAI) based on a previously published scale of zero to 4 for any parameter; normal status should remain as zero and highest activity as 9 [60, 61].

### Sacrifice

Dissection and tumor excision were done when tumor size reached 1cm^3^. The animals were anesthetized by an overdose of Forane (Isoflurane), the abdominal cavity was exposed and a macroscopic assessment of the inflammatory status was performed according to an already published scale [60].

Biopsies of the descending colon (DC), small intestine, liver and kidneys were collected. The tissues obtained were either transferred into labeled aliquots, snap-frozen in liquid nitrogen, and kept at -80°C for further molecular analysis or were kept in 10% formaldehyde to be processed with paraffin for routine light microscopy and histology analysis according to previously reported procedures [60].

### Real time RT-PCR

The total RNA of the tissues was extracted using an RNeasy mini-kit (Qiagen Ltd., Crawley, United Kingdom). RNA quantity and purity were assessed using NanoDrop ND-1000 spectrophotometer (Wilmington, NC). M-MLV Reverse Transcriptase buffer pack (Promega, Lyon, France) was used for reverse transcription. Primers were designed for the determination of the following gene expression: mTORC1, AMPK, IL-6, IL-3, and TNF-ɑ. GAPDH was used as an internal control. The amplification was monitored with StepOnePlus PCR System (AB Applied Biosystems, Villebon-sur-Yvette, France) using GoTaq qPCR Master Mix (Promega, Charbonnieres Les Bains, France) according to manufacturer's instructions. Samples were run in triplicate, relative abundance of each target was normalized to GAPDH expression and gene regulation was determined by the quantitation-comparative ΔΔCT method [62].

### Western blot

Protein extraction and quantification were performed using previously established protocols. The extracted proteins were separated by gel electrophoresis and were transferred onto nitrocellulose membranes. The membranes were blocked with 5% bovine serum albumin in Tris-buffered saline and probed with primary antibodies specific for phospho-mTOR, and mTOR (all from Cell Signaling Technology) and GAPDH. Horseradish peroxidase-conjugated secondary antibodies and the ECL detection kit (Bio-Rad) were used for the detection of specific proteins. Bands were quantified and normalized to the signal generated from GAPDH.

### ROS detection by DHE staining

Frozen sections, from frozen tissue stored at (-80), were prepared. The tissue was demarcated with a solvent resistant pen. DHE solution was prepared and dispensed over the tissue and the slides placed for 30 min at 37°C. Then the DHE residues were removed, slides counterstained with DAPI, colversliped and stored at 4°C (light sensitive) until microscopic evaluation and quantification using Zen software. One way ANOVA: to compare between all of the groups and T-test: to compare between two groups were done [60].

### Histology

Tissue preparation for light microscopy was performed according to routine procedures and protocols already established in the laboratory [60]. The histological alterations were assessed using a previously published scale illustrated in Table 2 [60]. Fields at 200 x magnification were photographed, evaluated and scored by 2 independent researchers. The scores of two independent observers were averaged. The histological grades (from “0 to 21”) indicating the numerical sum of scoring criteria were divided by 7 (the number of criteria), averaged to obtain a maximum average of 3, computed and represented with matching standard error of the mean [60].

**Table 2:** Criteria for microscopic grading of experimental chronic colitis [60]

Histologic grading				
Feature	0	1	2	3
**Abnormalities of mucosal architecture**	None (Normal)	Mild or focal, not exceeding lamina propria	Moderate, not exceeding the submucosa	Severe & diffuse, exceeding the submucosa
**Crypt abnormalities**	None	Mild atrophy	Moderate atrophy, Branched crypts	Severe atrophy, branched crypts, cryptitis, crypt abscess
**Inflammatory cell infiltration**	Normal	Scattered cells	Moderate or confluent cells	Massive infiltration of cells
**Vascular dilatation**	Normal	Mild dilatation (localized)	Moderate dilatation of several blood vessels	Severe generalized dilatation of blood vessels
**Edema**	None	Low level limited to villi	In the submucosa	All over the section
**Mast cells**	Normal	Three cells clustered in submucosa	Clusters of > 3 cells in the submucosa	Clusters in submucosa and serosa

### Mast cells count

The evaluation of mast cell count was performed by two different observers according to previously reported criteria on slides stained with Toluidine Blue (TB) [60].

### Statistical analysis

Statistics were conducted using the analysis of t-test and ANOVA to compare each experimental group to the corresponding controls using the STAT3 software. Significance was determined as probability (p) <0.05.

### Limitations of the study

This study has several limitations. First, a small number of animals per treatment group was used (n=6), especially in the probiotics treated mice, whereby, more controls groups could have been also included. Moreover, this study only assessed the response of male NOD/SCIDs mice, it would have been better if the 2 genders were part of the study.

Another major limitation of this CRC model is the NOD/SCIDs mice, they have a compromised immune system leading to the loss of the complex interactions between tumor and host. Thus, they may not represent the behavior of naturally occurring cancers in humans.

Another restraint is the genetic and epigenetic changes which may occur in the tumor cells during culture and implantation, despite the fact that the cells were in early stages of culture.

Therefore, future studies assessing the effects of rapamycin, metformin and probiotics should be conducted on a larger number of animals from both genders. Clinical studies are also required to demonstrate the beneficial effects of these treatments on patients and to elucidate the safety and correct regimens for the prevention and management of CRC.
