# Correction: A novel therapeutic approach to colorectal cancer in diabetes: role of metformin and rapamycin

**DOI:** 10.18632/oncotarget.27298

**Published:** 2019-11-05

**Authors:** Alice Gerges Geagea, Manfredi Rizzo, Abdo Jurjus, Francesco Cappello, Angelo Leone, Giovanni Tomasello, Céline Gracia, Sahar Al Kattar, Liliane Massaad-Massade, Assaad Eid

**Affiliations:** ^1^ Department of Internal Medicine, University of Palermo, Palermo, Italy; ^2^ Department of Anatomy, Cell Biology and Physiological Sciences, Faculty of Medicine, American University of Beirut, Beirut, Lebanon; ^3^ Equipe Nouvelles Thérapies Anticancéreuses, UMR8203 CNRS, Gustave Roussy, Villejuif, France; ^4^ Department of Biomedicine, Neurosciences and Advanced Diagnosis, School Of Medicine of Palermo, Palermo, Italy

**This article has been corrected:** Due to errors in image preparation, two panels in Figures 4 and 5 were accidentally duplicated. The corrected Figures 4 and 5 are shown below. The authors declare that these corrections do not change the results or conclusions of this paper.

Original article: Oncotarget. 2019; 10:1284–1305. 1284-1305. https://doi.org/10.18632/oncotarget.26641

**Figure 4:**
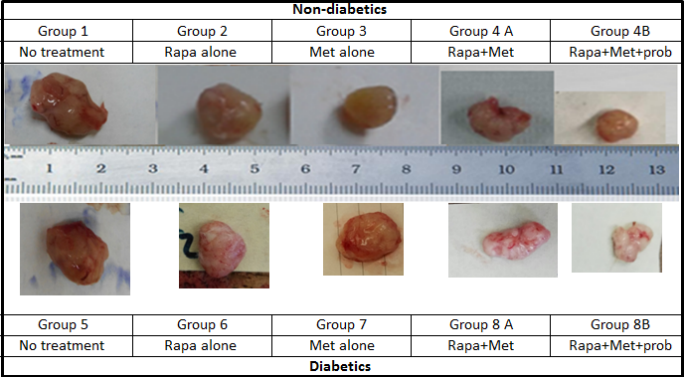
Prototype of tumors upon sacrifice, formed in non-diabetic and diabetic mice treated with rapamycin, metformin and their combination with probiotics. Note the difference in tumor size in the different groups; animals from groups 4B and 8B treated with rapamycin and metformin in combination with probiotics had significantly smaller tumor size when compared to groups treated with Met alone, rapamycin alone or untreated animals.

**Figure 5:**
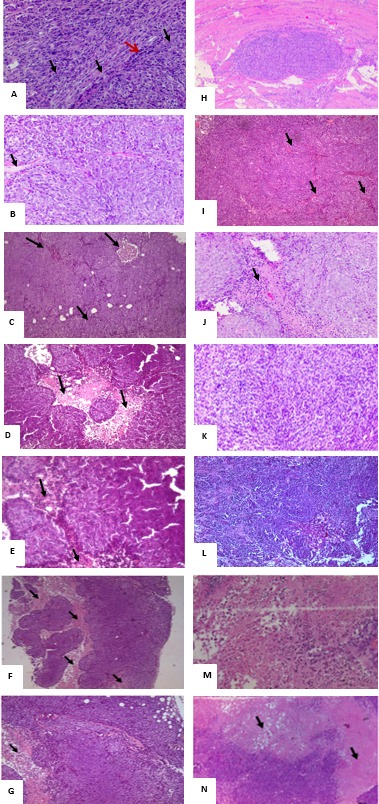
H&E histological examination of the representative HCT116 xenograft tumors in the different groups. (**A**) a 200x magnification of a tumor section in non-diabetic, injected with HCT116 cells and non-treated showing high cellular density, vascularization (black arrows), and tumor cells surrounded by a remarkable infiltration of inflammatory cells (red arrows). (**B**) a 20x magnification showing some necrotic areas in the tumor (Black arrows) in non-diabetics, rapamycin alone (G2) (**C**) 20x magnification showing some necrotic areas in the tumor (Black arrows) in non-diabetics metformin alone (**D**) 4x and (**E**) 200x magnification show large necrotic areas in the tumor section with low cell density (black arrows) in non-diabetics treated with metformin combined to rapamycin. (**F**) 40x and (**G**) 200x magnification showing necrotic areas (Black arrows), along with a lower density of the cells in diabetic treated with metformin, rapamycin combined with probiotics (G4B). Note that all tumors from 5 animals in the same group showed similar morphology. (**H**) Whole view of a well-demarcated tumor formed with a scanty fibrous capsule and a moderately produced connective tissue in diabetic non-treated animals. Note the sheet-like proliferation showing growth of solid tumor cells. (**I**) 200x magnification of the tumor section in G5, note the high density of the cells along with increase in vascularity (black arrows) (**J**) 200x magnification of a tumor section, showing a moderate cells density along with an increase in vascularity (black arrows) in diabetics treated with rapamycin alone (G6). (**K**) 200x magnification of a tumor section, note the moderate density of the cells in diabetics treated with metformin alone (G7). (**L**) 200x magnification of tumor section from diabetic mice treated with metformin and rapamycin showing a lesser density of the cells than either alone (**M**) 200x and (**N**) 20x magnification of tumor section from diabetic mice with the triple therapy showing necrotic areas (Black arrows), along with a significant decrease in cellular density.

